# Selective herbicide safening in dicot plants: a case study in *Arabidopsis*

**DOI:** 10.3389/fpls.2023.1335764

**Published:** 2024-01-15

**Authors:** Gabriela Pingarron-Cardenas, Nawaporn Onkokesung, Alina Goldberg-Cavalleri, Gudrun Lange, Jan Dittgen, Robert Edwards

**Affiliations:** ^1^ Agriculture, School of Natural and Environmental Sciences, Newcastle University, Newcastle Upon Tyne, United Kingdom; ^2^ Bayer Aktiengesellschaft (AG), Crop Science Division, Computational Life Sciences, Frankfurt, Germany; ^3^ Bayer Aktiengesellschaft (AG), Crop Science Division, Weed Control Research, Frankfurt, Germany

**Keywords:** cereal, flufenacet, glutathione transferase, herbicide detoxification, isoxadifen-ethyl

## Abstract

Safeners are agrochemicals co-applied with herbicides that facilitate selective control of weeds by protecting monocot crops from chemical injury through enhancing the expression of detoxifying enzymes such as glutathione transferases (GSTs). Even though the application of safeners causes the induction of genes encoding GSTs in model dicots such as *Arabidopsis thaliana*, safeners do not protect broadleaf crops from herbicide injury. In this study, we proposed that the localized induction of *Arabidopsis* GSTs and the fundamental differences in their detoxifying activity between dicot and monocot species, underpin the failure of safeners to protect *Arabidopsis* from herbicide toxicity. Using the herbicide safener, isoxadifen-ethyl, we showed that three tau (U) family GSTs namely *AtGSTU7*, *AtGSTU19* and *AtGSTU24* were induced with different magnitude by isoxadifen treatment in root and rosette tissues. The higher magnitude of inducibility of these *AtGSTUs* in the root tissues coincided with the enhanced metabolism of flufenacet, a herbicide that is active in root tissue, protecting *Arabidopsis* plants from chemical injury. Assay of the recombinant enzyme activities and the significant reduction in flufenacet metabolism determined in the T-DNA insertion mutant of *AtGSTU7* (*gstu7*) in *Arabidopsis* plants identified an important function for *At*GSTU7 protein in flufenacet detoxification. *In-silico* structural modeling of *At*GSTU*7*, suggested the unique high activity of this enzyme toward flufenacet was due to a less constrained active site compared to *At*GSTU19 and *At*GSTU24. We demonstrate here that it is possible to induce herbicide detoxification in dicotyledonous plants by safener treatment, albeit with this activity being restricted to very specific combinations of herbicide chemistry, and the localized induction of enzymes with specific detoxifying activities.

## Introduction

Herbicide safeners are an important class of agrochemicals used to protect cereals from chemical injury by enhancing herbicide tolerance in the crop, but not in competing weeds (Kraehmer et al., 2014). Typically, safeners are co-applied with partner herbicides in well-defined combinations to enhance selectivity in weed control in specific cereal crop species (Giannakopoulos et al., 2020). Safener activity in *Oryza sativa* (rice), *Hordeum vulgare* (barley), *Triticum aestivum* (wheat), *Zea mays* (maize), and *Sorghum bicolor* (sorghum) is typically linked to the enhanced expression of herbicide-metabolising enzymes, notably cytochromes P450 monooxygenases (CYPs) and glutathione transferases (GSTs), that catalyse primary detoxification reactions (Riechers et al., 2010). This enhancement follows the increased transcript expression of the respective genes, with multiple GSTs and CYPs induced, each with its own spectrum of detoxifying activities (Brazier-Hicks et al., 2018).

In contrast, safeners have not been commercially developed to enhance herbicide tolerance in dicotyledonous crops (Kraehmer et al., 2014). While there are references to limited efficacy of safeners in protecting horticultural species such as tomato (Castrol et al., 2020), studies with broadleaf arable crops such as soybean have shown no enhanced herbicide detoxification, nor agronomically useful protective effects (Andrew et al., 2005). Extending applications for safening into broad-leaf crops could potentially allow existing chemistries to be used in new applications as selective herbicides. As such, understanding the underpinning biology for this difference in safener responsiveness in dicots and monocots is of great interest. As a model species, *Arabidopsis thaliana* undergoes extensive induction of multiple genes, including those classically involved in herbicide detoxification, when exposed to safeners such as isoxadifen-ethyl (IDF) or fenclorim (Brazier-Hicks et al., 2008, Behringer et al., 2011). Studies in *Arabidopsis* transformed with the promoter of a safener responsive lambda GST from maize (In2-1) linked to a reporter gene have further demonstrated a conservation in associated signaling pathways (de Veylder et al., 1997). However, unlike cereals, this safener-responsive gene induction does not protect *Arabidopsis* plants from herbicide toxicity (DeRidder et al., 2002; DeRidder and Goldsbrough, 2006).

Two main hypotheses have been proposed to explain the disconnect between safener action at the level of gene expression without any apparent enhancement in herbicide tolerance in *Arabidopsis* and other dicot species. The first hypothesis is that unlike the case in cereals, the induction of detoxifying enzymes such as GSTs in dicotyledonous species is restricted to tissues which are not targeted by herbicides. The study by DeRidder and Goldsbrough (2006) reported that *AtGSTU19*, a tau (U) class GST in *Arabidopsis* which was naturally expressed in root tissues, was significantly induced in roots, but not in hypocotyls or leaf tissues, after treatment with the safeners benoxacor and fenclorim, respectively. The localized induction of *AtGSTU19* in roots coincided with failure to induce protective effects when the safeners were applied with chloroacetanilides (alachlor and *S-*metolachlor), herbicides that are most active at the growing points, notably shoot meristems. These results suggest a potential link between localized induction of GSTs and the lack of protective effects against specific herbicides. A second hypothesis proposes that while safeners can generally induce enhanced expression of genes involved in detoxification, this induction may not translate into an increase in the respective functional xenobiotic-metabolizing enzymes. However, this hypothesis can be considered unlikely based on available evidence. For instance, it has been shown in *Arabidopsis* that exposure to the safeners benoxacor and fenclorim led to enhanced expression of tau (U) and phi (F) class GSTs as well as enhanced activity toward model GST substrates such as 1-chloro-2,4-dinitrobenzene (CDNB) (DeRidder et al., 2002; DeRidder and Goldsbrough, 2006; Brazier-Hicks et al., 2008; Skipsey et al., 2011). As an extension of this observation, it was reported that fenclorim which is itself metabolized by *S*-glutathionylation catalysed by GSTs, actively promotes its own detoxification when fed to *Arabidopsis* through its safener action (Skipsey et al., 2011).

The aim of this work is to test our new hypothesis in order to explain the failure of safeners to induce effective safening in *Arabidopsis*, a model dicot species. In this study we have used the isoxazoline safener, isoxadifen-ethyl (IDF) which is typically used in cereals to enhance selectivity through increasing the rate of herbicide detoxification by enzymes including GSTs (Kraehmer et al., 2014). The approach adopted has been to establish whether there is a link between enhanced metabolism and tolerance to herbicides detoxified by GSTs, since this safener is known to induce multiple GST genes in *Arabidopsis* when applied as a single chemical treatment (Behringer et al., 2011). However, IDF remains inactive on dicot crops as a safener when co-applied with herbicides under normal agricultural practise (tank-mix), prompting us to speculate that both the confinement of activity to specific tissues, as well as the timing of treatment with safener vs. herbicide may explain the inactivity of safeners in dicots. Instead, we conjectured that the right timing of safener application may be able to induce effective herbicide detoxification in specific tissues of dicots. *S-*metolachlor (*S*-MOC), a chloroacetamide herbicide that is normally used with a range of safeners, and flufenacet, an oxyacetanilide herbicide that does not require the use of safeners (Ducker et al., 2019a; Ducker et al., 2019b) were selected for this study. Both herbicides have been confirmed to be detoxified by GSTs (Ducker et al., 2019a; Ducker et al., 2019b; Strom et al., 2021; Parcharidou et al., 2023). In our experimental approach we first looked for effects of IDF on localized transcript expression of GSTs in root culture and rosette tissues of *Arabidopsis.* The functional characterization of recombinant proteins was then used to provide evidence for the spectrum of detoxification activity toward *S*-MOC and flufenacet by selected safener inducible *At*GSTUs. The objective of this study has been to establish a fundamental understanding of the effects of herbicide safeners in dicot species which could lead to novel routes to ‘safening’ and expand the application of selective herbicides in broad leaf crop species.

## Materials and methods

### Chemicals

The herbicides *S-*metolachlor (*S*-MOC) and flufenacet (FFA) and the safener isoxadifen-ethyl (IDF) were purchased from Sigma Aldrich (UK). 100 mM stock solutions were prepared in dimethyl sulfoxide (DMSO). The herbicide glutathione conjugates were prepared by mixing either *S*-MOC (50 µmol) or FFA (17.5 µmol) with 50 µmol of glutathione (GSH). The reaction mixtures were adjusted to pH 8.5 with Tris (*S*-MOC), or to pH 9.0 with triethylamine (FFA) after mixing with an equal volume of acetonitrile: ethanol (1:1) respectively. The reactions were incubated at 28°C for 48 h. The reactions were analyzed on Acquity UPLC system coupled with Quadrupole time-of-flight mass spectrometry (QTOF-MS) (UPLC-qTOF MS, Waters, UK) and the authenticity of the reaction products was confirmed by accurate mass (*m/z*) of each compound (**Supplementary Table S1**).

### Plant materials

*Arabidopsis thaliana* ecotype Columbia-0 (Col-0, WT) and the T-DNA-insertion mutant of *AtGSTU7* (*gstu7*, SALK 086642C) were obtained from the European Arabidopsis Stock Centre (NASC, Nottingham, U.K.). *Arabidopsis* seeds were surface sterilized in 5% (v/v) sodium hypochlorite containing 0.1% (v/v) Tween 20 for 5 min and washed (5-time) with sterile water. Seeds were distributed on Murashige and Skoog (MS) media containing 3% (w/v) sucrose and stratified at 4°C in the dark for 3 d. Plates were transferred to growth cabinets maintained at 12h/12h (light/dark) photoperiod, and 21°C/16°C respectively. Seedlings (14-d after germination) were then transferred to plastic pots (5 cm) containing John-Innes No.2 compost and maintained under identical growth conditions.

For root tissues, 5d-old seedlings were transferred to conical flasks containing 100 mL Gamborg´s B5 liquid medium and incubated on an orbital shaker (120 rpm) at 25°C in the dark. After 14 d, seedling cultures comprising roots and etiolated hypocotyls were used for experiments.

### Herbicide and safener treatment

For herbicide toxicity assays, 3-week-old (rosette stage) soil-grown *Arabidopsis* plants were treated with IDF and either *S*-MOC or FFA, using the following combinations: 100 µM IDF alone, 100 µM herbicide alone or a 24 h pre-treatment with 100 µM IDF followed by 100 µM herbicide. Controls consisted of treating with the carrier solvent, 0.1% (v/v) DMSO, alone. Chemical treatments were applied by hand spraying plants to ‘run-off’. Herbicide injury and rosette fresh mass (FM) were then assessed in individual plant at 14 d after treatment. Three to five individual plants (n = 3-5) were used for each treatment.

For herbicide metabolism and transcript expression studies, excised roots of 14d old root cultures, or excised rosettes of 3-week-old soil-grown *Arabidopsis* plants were pre-treated with either IDF, or an equivalent volume of DMSO for 24 h prior to the addition of herbicides, with all chemical treatments used at a final concentration of 100 µM. Root or rosette tissues were removed from the solutions at 1 h, 6 h, 24 h and 48 h and were briefly rinsed in acetonitrile. Tissue samples were blotted on paper towels to remove excess treatment solution, weighed, flash frozen in liquid nitrogen and stored at -80°C until analysis. Treatment media were also collected for analysis of parent herbicides and their metabolites. Two individual rosettes were combined to make 1 biological replicate, and 3 biological replicates (n = 3) were used for each treatment. For root tissues, roots from 7 seedlings were combined to make 1 biological replicate, and 3 biological replicates (n = 3) were used per treatment.

### Herbicide metabolite analysis

Frozen tissue samples were pulverised with a pestle and mortar and 3 g of root tissue, or 1 g of rosette tissue were extracted with 5 (v/w) of 80% (v/v) methanol for 24 h at 4°C. Treatment media samples were diluted with 5 volumes of methanol. All samples were centrifuged (3000g, 3 min), and supernatants were collected for analysis. Samples (5 µL) were injected to an UPLC-qTOF MS operating in positive ion mode with electrospray ionization. Samples were separated on BEH C18 column (130Å, 1.7 µm, 3 mm X 100 mm, Waters, UK) using a 3 min gradient (0 to 100%) of mobile phases A (0.1% (v/v) formic acid) and B (0.1% (v/v) formic acid in acetonitrile) at a flow rate of 0.4 mL min^-1^. Instrument control and data analysis was performed using MassLynx software version 4.1 (Waters, UK). The herbicide metabolites were determined based on calculated accurate mass (*m/z*). The parent herbicide molecules and the respective glutathione conjugates were quantified using external standard curves prepared as described above.

### Glutathione transferase enzyme assays

Frozen tissue samples were pulverized in liquid nitrogen and extracted in 5:1 (v/w) of 50 mM Tris-HCl (pH 7.5) containing 2 mM EDTA, 1 mM dithiothreitol (DTT) and 2% (w/v) polyvinylpolypyrrolidone. The mixture was filtered through Miracloth and centrifuged at 11000 g, 4°C for 20 min. Supernatants were collected and the protein concentration was determined by Bradford assay followed the manufacturer’s protocol (BioRad, U.K). Glutathione transferase (GST) activity toward 1-chloro-2,4-dinitrobenzene (CDNB) was determined by spectrophotometry as described previously (Dixon et al., 1998). GST activity toward FFA and *S-*MOC was performed by mixing 20 µL of 1 mM Tris-HCl (pH 7.5), 10 µL of 20 mg bovine serum albumin (BSA) and 50 µg of protein extract with 170 µL of water. The reactions were incubated at 30°C for 3 min before 10 µL of 10 mM herbicides and 20 µL of 10 mM glutathione were added. After 40 min incubation, the reaction was stopped by adding 10 µL of 3M HCl and samples were stored at -20°C until analysis by UPLC-qTOF MS.

### Transcript expression analysis

Total RNA was extracted from finely ground frozen tissues using the TRIzol Reagent (Invitrogen) protocol (Skipsey et al., 2011). RNA quality and concentration were determined on a TapeStation system (Agilent, UK) prior to cDNA synthesis from 1 µg of total RNA by SuperScript II Reverse Transcriptase system (Invitrogen, Fisher Scientific UK) using the manufacturer’s protocol. cDNA samples were diluted in equal volume of nuclease free water and stored at -20°C until analysis.

Quantitative real-time PCR (qPCR) was performed on a LightCycler 480 (Roche Diagnostics, UK) in a total volume of 20 µL containing 2 µL of cDNA, 10 µL Luna SYBR green qPCR master mix (New England Biolab, UK), and 0.5 µL of 10 µM forward and reverse specific primers. The primers were designed by Primer3 online software (version 4.1.0) and are listed in **Supplementary Table S2**. The reactions were run using a 3-step programme including melting curve analysis; preincubation at 95°C for 90 sec; amplification for 40 cycles (95°C for 15 sec, 58°C for 15 s and 72°C for 15 sec; followed by melting curve analysis from 65°C to 95°C). For normalization, specific primers for the SAND family gene (MONESIN SENSITIVITY 1; AT2G28390) were used (Czechowski et al., 2005). All reactions were performed with 3 biological replicates (n =3), relative gene expression was calculated as described in Pfaffl (2001).

### Heterologous expression and analysis of recombinant Arabidopsis GSTs

The pET-STRP3 expression vectors containing coding sequences of *AtGSTU7, AtGSTU19* and *AtGSTU24* were available from a previous study (Dixon et al., 2009). The recombinant proteins were expressed in *Escherichia coli* as Strep-tagged fusion proteins. Recombinant (r) proteins were recovered from the lysed *E. coli* using Strep-Tactin affinity chromatography at 24h after induced protein expression by isopropyl β-d-1-thiogalactopyranoside (IPTG). The proteins were quantified and prepared for assay as described (Dixon et al., 2009). The purified enzymes were assayed for GST activity toward FFA and *S*-MOC by UPLC-qTOF MS, with the respective kinetic constants K_m_ and V_max_ calculated by fitting the Michaelis-Menten equation to the initial velocity results determined over a range of substrate concentrations using non-linear regression (R-software).

### Phylogenetic analysis and *in-silico* protein modeling

For phylogenetic analysis, *Arabidopsis* GSTU sequences were extracted from the database (www.arabidopsis.org) and compared with the sequences of tau GSTs sequences from maize, wheat and blackgrass (www.ncbi.org). These GSTs from monocot species were selected based on their activity toward herbicides. Sequences were aligned and trimmed as described previously (Goldberg-Cavalleri et al., 2023). Automatic model selection mode was employed (Bayesian information Criterion selecting the model LG+G4+I+F), with branch support values calculated by Ultrafast bootstrap approximation. The tree was edited with the iTOL tool (https://itol.embl.de/) as described (Lutunic and Bork, 2016).

For *in silico* protein modeling, GST sequences were aligned using Blast with limited manual curations of the sequence alignment made after inspecting the AlphaFold2 (AF2) predicted models. For adduct fitting and docking studies, the editing module of SeeSAR and the HYDE scoring from BioSolveIT was employed (Reulecke et al., 2008).

### Statistical analysis

Herbicide metabolism data obtained from UPLC-qTOF MS, enzymatic activity and relative transcript expression data were compared between treatments using one-way ANOVA followed by the Tukey *post-hoc* test or the results were compared between WT and *gstu7* Arabidopsis mutant plants using Student’s *t*-test. SPSS software version 27.0 was used for all analysis.

## Results

### The differential induction of GSTs transcript in *Arabidopsis* root and rosette tissues

**Figure 1:**
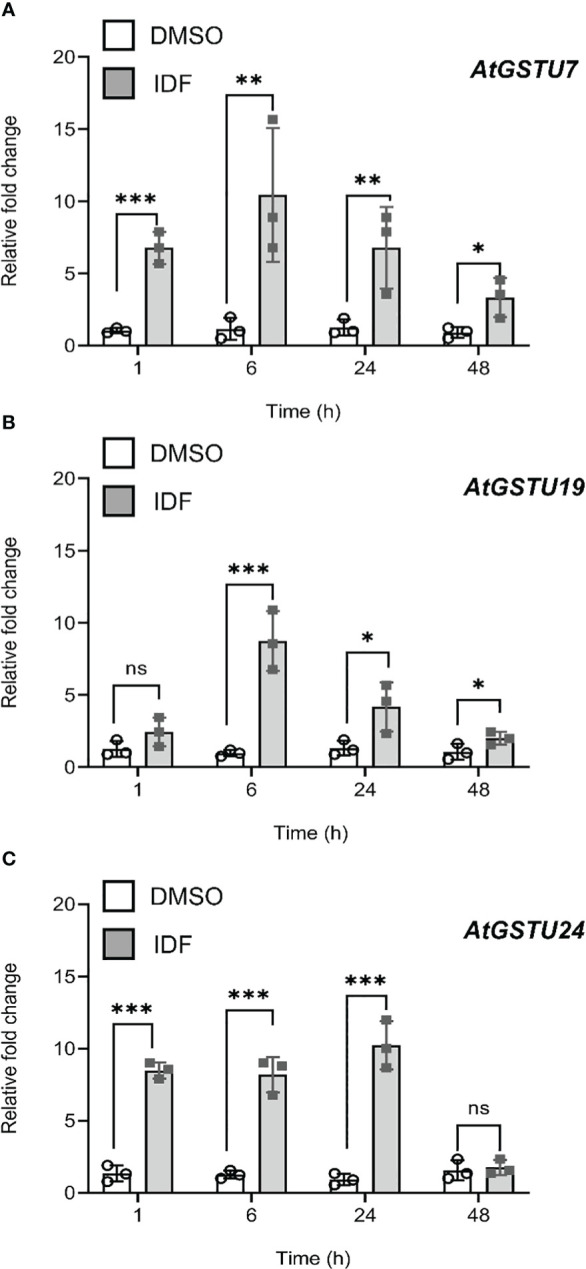
Isoxadifen treatment induced transcript expression of *AtGSTUs* in *Arabidopsis* root tissues. Root tissues from *Arabidopsis* seedling grown in the dark were treated with carrier solvent alone (DMSO) or isoxadifen (IDF). The relative fold change of **(A)** *AtGSTU7*, **(B)** *AtGSTU19* and **(C)** *AtGSTU24* were determined at designated time points. Each bar represents an average fold change of three biological replicates (means ± SD, n= 3) and the symbols in each bar represent fold change of the individual sample. The relative fold changes were compared between the treatment using Student’s *t*-test; asterisks indicate significant differences; *p ≤ 0.05; **p ≤ 0.01; ***p ≤ 0.001.

Behringer et al. (2011) reported that treatment of *Arabidopsis* rosette tissues with the herbicide safener IDF significantly induced transcript expression of genes associated with xenobiotic detoxification, including 13 GSTs. Similarly, treatment of *Arabidopsis* with the safener benoxacor enhanced the expression of GSTs in the foliage, but failed to provide functional safening against chloroacetamide herbicides such as alachlor, or *S-*metolachlor (*S*-MOC) (DeRidder and Goldsbrough, 2006). These observations inferred that induction of GSTs in *Arabidopsis* shoot tissue could not promote safening. However, the potential for safening via the root tissue using herbicides that are detoxified by inducible GSTs but target root meristems, rather than hypocotyls, such as FFA has not been reported. To probe this paradigm, we quantified the relative expression of the nine IDF-inducible *Arabidopsis* GSTs (*AtGSTs)* identified by Behringer et al. (2011) in *Arabidopsis* root cultures. These *AtGST* candidates included members of the tau (U), phi (F) and Lambda (L) families; classes of GSTs which have been previously associated with protective activities against herbicides. Over the period of IDF-treatment of the root tissues, *AtGSTU7*, *AtGSTU19* and *AtGSTU24* showed the highest induction of transcript expression (~10-15 fold) (**Figures 1A–C**). The expression of *AtGSTU10, AtGSTU11*, and *AtGSTF8* were also induced by IDF treatment, albeit at a lower magnitude (~3-5 fold) (**Supplementary Figures S1A–C**). In contrast to previous studies using foliar tissue (Behringer et al., 2011), the expression of *AtGSTL1*, *AtGSTF2* and *AtGSTU26* remained unchanged in the roots as compared to the solvent control (DMSO) treatment over 48 h (**Supplementary Figures S1D–F**).

To enable us to examine whether the magnitude of localized induction of *AtGSTU7, AtGSTU19 and AtGSTU24* in root and rosette tissues could enhance the metabolism of the root-active herbicide flufenacet, we quantified the expression of these *AtGSTUs* in roots and rosette tissues over a 24 h period following treatment. To gain more information on the expression patterns of these *AtGSTUs*, we devised 5 treatment conditions including DMSO alone, IDF alone, flufenacet (FFA) herbicide alone, a co-application of IDF and FFA and pre-treatment of IDF for 24 h before FFA application (**Supplementary Figure S2**). These treatment conditions were used because the information on the impact of herbicide safeners on the expression of detoxifying genes in *Arabidopsis* is normally derived from comparing expression levels between solvent controls and safener treatments (DeRidder et al., 2002; DeRidder and Goldsbrough, 2006; Brazier-Hicks et al., 2008; Skipsey et al., 2011). As safeners can either be used as pre-treaments or as co-applications with herbicides, it was of interest to examine the impact of different timings and combinations of safener vs. herbicide applications on *AtGSTU* expression levels. Treatments therefore included both a co-application of IDF and FFA, together with an IDF pre-treatment 24 h before FFA application.

**Figure 2:**
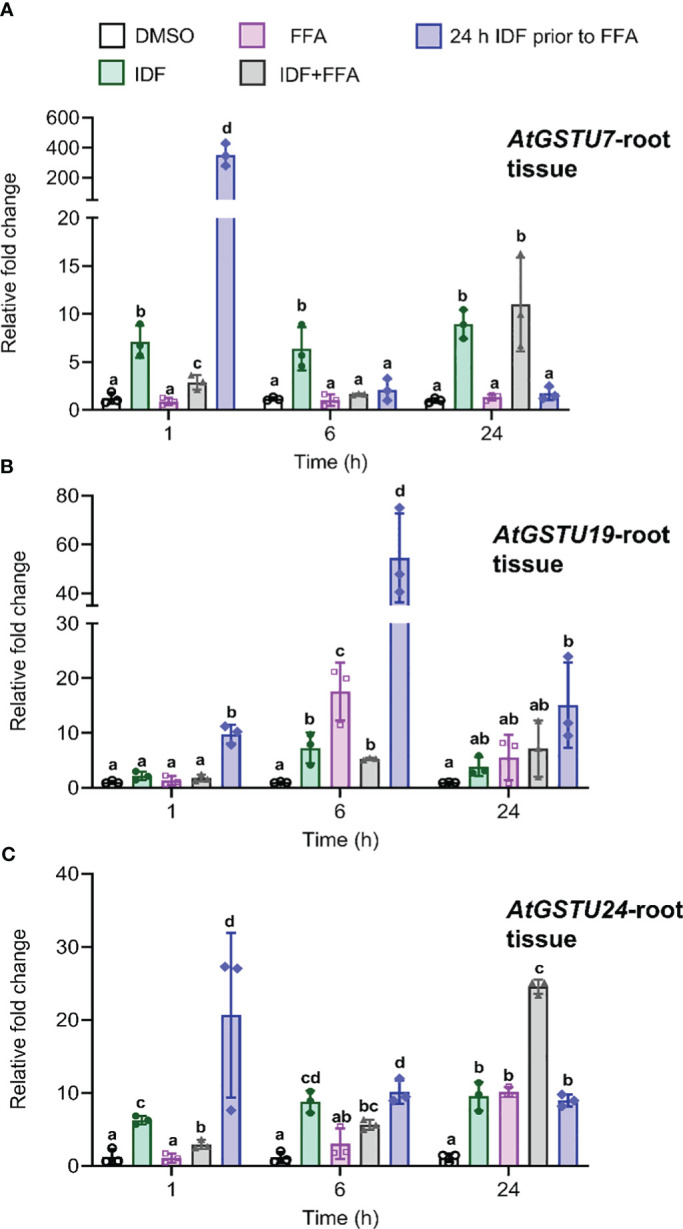
The differential effects on transcript expression of *AtGSTUs* in *Arabidopsis* root tissues treatment with isoxafiden, flufenacet, co-application of isoxadifen and flufenacet, or pre-treatment with isoxadifen followed by flufenacet. Root tissues from Arabidopsis seedlings grown in the dark were treated with carrier solvent alone (DMSO), isoxadifen (IDF), flufenacet (FFA), co-application of IDF and FFA or pre-treatment of IDF for 24 h prior to application of FFA. The relative fold changes of **(A)** *AtGSTU7*, **(B)** *AtGSTU19* and **(C)** *AtGSTU24* were determined at designated time points. Each bar represents an average fold change of three biological replicates (means ± SD, n= 3) and the symbols in each bar represent fold change of the individual sample. The relative fold changes were compared among the treatment using one-way ANOVA followed by turkey’s *Posthoc* test, the different letter indicate significant differences; p ≤ 0.05.

In root tissue, the pre-treatment of IDF for 24 h before FFA treatment led to an earlier induction (1 h after FFA application) of *AtGSTU7*, *AtGSTU19*, and *AtGSTU24* as compared to the combined treatment of IDF+FFA, where maximal enhancement was observed at 6 h, or 24 h after treatment respectively (**Figure 2**). *AtGSTU7* was the most responsive gene, with its transcripts transiently enhanced over 300-fold by the 24 h pre-treatment with IDF followed by FFA (**Figure 2A**). The pre-treatment regime also invoked 60-fold and 40-fold increases in *AtGSTU19* and *AtGSTU24* respectively in root tissues (**Figures 2B,C**). It is interesting that this synergism resulted in a very transient induction of the three *At*GSTs in the IDF pre-treated roots, with *AtGSTU7* and *AtGSTU19* being maximally enhanced after just 1 h following FFA treatment, and *AtGSTU24* after 6 h. In contrast, the treatment of root tissues with IDF or FFA alone, or by co-application of the two compounds tended to result in a more modest, prolonged induction of the three GSTs (**Figure 2**).

In the rosette tissue, a lower magnitude of induction levels of *AtGSTU7*, *AtGSTU19* compared to root tissues were observed over the 24 h period (**Supplementary Figure S3**). However, *AtGSTU24* was transiently enhanced over 100-fold in rosettes pre-treated for 24 h with IDF followed by a 1 h exposure to FFA (**Supplementary Figure 3C**). While the expression pattern of the three *AtGSTUs* was similar in rosette and root tissues, with expression being enhanced by exposure to IDF, but not after treatment with FFA alone, the magnitude of their induction was clearly much lower in shoot tissue than in root tissue. These results support our hypothesis that IDF treatment can elicit localized induction of *AtGTSUs* in root and rosette tissues at different orders of magnitude. It then followed, that if these *AtGSTUs* are involved in FFA detoxification, we should detect enhanced metabolism of FFA in root but not in rosette tissues.

### Enhanced GST activity toward flufenacet in root tissues of *Arabidopsis*

**Figure 3:**
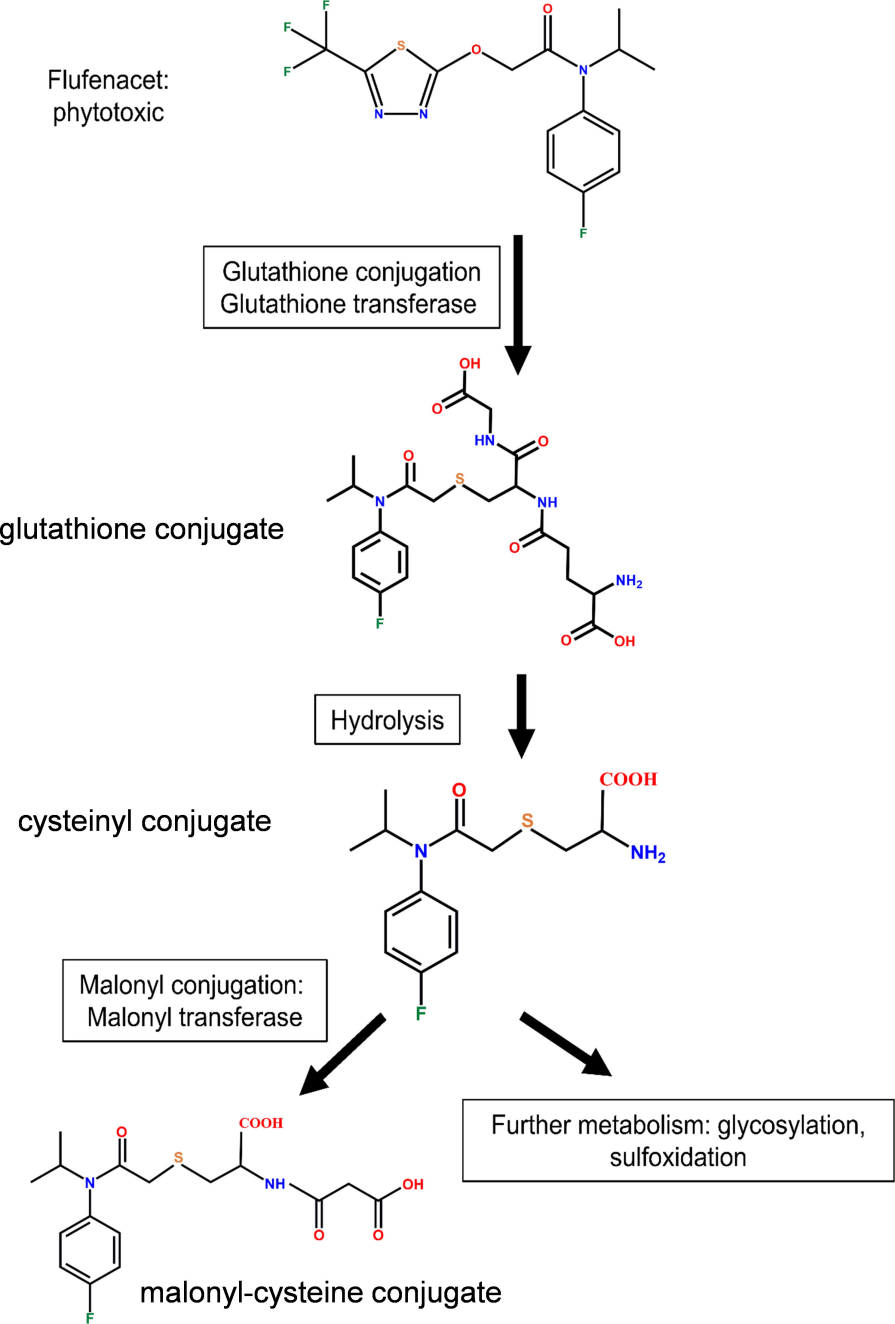
The detoxification of flufenacet by GST catalysed *S-*glutathionylation in plants.

FFA is known to be metabolized in plants by GSTs to produce the respective glutathione conjugates (**Figure 3**), which are less toxic than the parent herbicide (Ducker et al., 2019a; Ducker et al., 2019b; Ducker et al., 2020). To determine whether the different magnitudes of localized induction of *AtGSTUs* in root and rosettes affected GST activity toward herbicides, the crude protein isolated from *Arabidopsis* rosettes and root tissues were assayed for GST activity toward FFA following exposure of plant tissues to the five treatment regimes (**Supplementary Figure S2**).

**Figure 4:**
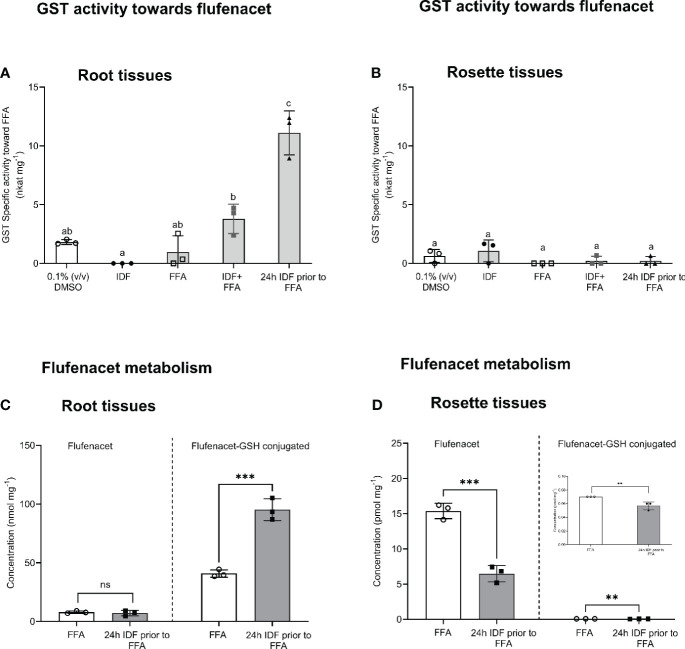
The effect of isoxadifen treatment on the glutathione conjugation of flufenacet and GST activity toward flufenacet in Arabidopsis. The formation of glutathione-conjugated herbicide (FFA-GSH conjugate) was quantified 24 h after treatments in **(A)** root, or **(B)** rosettes tissues. The concentrations of flufenacet and FFA-GSH conjugate were quantified using external standard curves of standards. The GST activity toward FFA was determined in crude extracts of **(C)** roots or **(D)** rosette tissues treated with agrochemicals for 24 **(h)** The insert **(D)** represents the activity toward FFA in rosette tissues. Each bar represents the average of three biological replicates (mean ± SD, n =3) of FFA, FFA-GSH conjugate concentrations or GST activity. The concentration of FFA and FFA-GSH conjugate were compared between samples treated with FFA with and without a 24 h pre-treatment with isoxadifen before FFA application, using Student’s t-test. Asterisks indicate significant differences; * p ≤ 0.05; ** p ≤ 0.01; *** p ≤ 0.001. The GST activities were compared using one-way ANOVA followed by turkey’s Posthoc test; different letters indicate significant differences (p ≤ 0.05).

In rosette tissue, GST activity from crude protein extract toward FFA was very low (< 1 nkat mg^-1^) and unaffected by the chemical treatments (**Figure 4B**). In the root tissue, GST activity was enhanced approximately 2-fold by co-application of IDF and FFA, as compared to the DMSO control. However, this difference was not deemed significant (**Figure 4A**, one-way ANOVA, p _(DMSO vs IDF+FFA)_ = 0.32). In contrast, pre-treatment with IDF for 24 h before application of FFA significantly induced GST activity (~5-fold) toward FFA (**Figure 4A**, one-way ANOVA, p _(24h pre IDF prior to FFA vs DMSO)_ = 0.001, p _(24h pre IDF prior to FFA vs co-application)_ = 0.001). The higher GST activity toward FFA in roots as compared with rosette tissue corresponded to the induction of *AtGSTU7, AtGSTU19* and *AtGSTU24* in the respective tissues (**Figures 2**, **4A, B**).

### Enhanced flufenacet metabolism in *Arabidopsis* root tissues after pre-treatment with isoxadifen

To correlate differences in localized GST transcript induction and associated enzyme activity with herbicide detoxification, the metabolism of FFA in root and rosette tissues was determined following IDF treatment. In addition, the apperance of GST-conjugates of *S*-metolachlor (*S*-MOC), a GST-detoxified herbicide active in hypocotyls, was determined for comparison. In each case herbicide metabolites were determined and quantified using UPLC-qTOF MS. To facilitate equivalence in treatment, plant tissues were treated by submerging rosettes or root tissues in aqueous solutions (Skipsey et al., 2011). Since the pre-treatments with IDF for 24 h led to significantly higher GST activity toward FFA compared to co-application, samples were pre-treated with, or without IDF (100 µM) for 24h before treatment with either 100 µM FFA, or 100 µM *S*-MOC. The herbicide metabolites were then assessed 24 h after treatment by reference to the known detoxification products of FFA (**Figure 3**) and *S*-MOC (**Supplementary Figure S4**). Using calculated accurate mass, four metabolites of FFA and four metabolites of *S*-MOC were identified in *Arabidopsis* root and rosette tissues (**Supplementary Figures 5**, **6**). Pre-treatment with IDF for 24 h before exposure to herbicides significantly increased the accumulation of metabolites of both FFA- and *S*-MOC in root tissues (**Supplementary Figures 5A**, **6A**). In contrast, herbicide metabolite formation in rosette tissues was significantly suppressed by pre-treatment with IDF (**Supplementary Figures 5B**, **6B**). With both herbicides, the respective glutathione (GSH) conjugates were the main detoxification products identified in both root and rosette tissues (**Supplementary Figures 5**, **6**). The GSH conjugates of both FFA and *S*-MOC were then quantified using external standard curves.

The levels of the herbicide *S*-MOC and its glutathionylated conjugate *S*-MOC-GSH were low in leaf and root tissue from both *S*-MOC treatment and *S*-MOC-IDF pre-treatment (pmol mg^-1^ FM). The formation of *S*-MOC GSH was significantly reduced in the rosettes by pre-treatment with IDF (**Supplementary Figure 7A**, Student’s *t*-test, p = 0.001), while in the roots, the level of *S*-MOC GSH in IDF pre-treatment were comparable to those treated with *S*-MOC alone (**Supplementary Figure 7B**, Student’s *t*-test, p = 0.18). From this we concluded that IDF was unable to enhance the glutathione conjugation of *S*-MOC in *Arabidopsis* root and rosette tissues. The lack of protective effect against *S*-MOC toxicity was in agreement with the results reported by DeRidder and Goldsbrough (2006) for Arabidopsis seedling treatments. With FFA, the levels of parent herbicide and FFA-GSH were around 1000-time lower (pmol mg^-1^ FM) in rosette tissues than those in root tissues (nmol mg^-1^ FM) (**Figures 4C, D**). This suggested that in our experimental system, both uptake and metabolism of FFA in rosette tissue was lower than in root tissues. The FFA-GSH level was significantly lower in rosette tissues pre-treated with IDF for 24 h than in rosette tissues treated with FFA alone (**Figure 4D**, Student’s *t*-test, p = 0.01). In contrast, the levels of FFA-GSH in root tissue was significantly enhanced (2.5-fold) in those samples pre-treated with IDF as compared to the FFA treatment alone (**Figure 4C**, Student’s *t*-test, p = 0.001). The significant enhancement of FFA metabolism in root tissues pre-treated with IDF therefore corresponded to the corresponding marked induction of *AtGSTUs*, notably of *At*GSTU7, *At*GSTU19 and *At*GSTU24.

### The protective effect of isoxadifen against flufenacet toxicity in *Arabidopsis*

**Figure 5:**
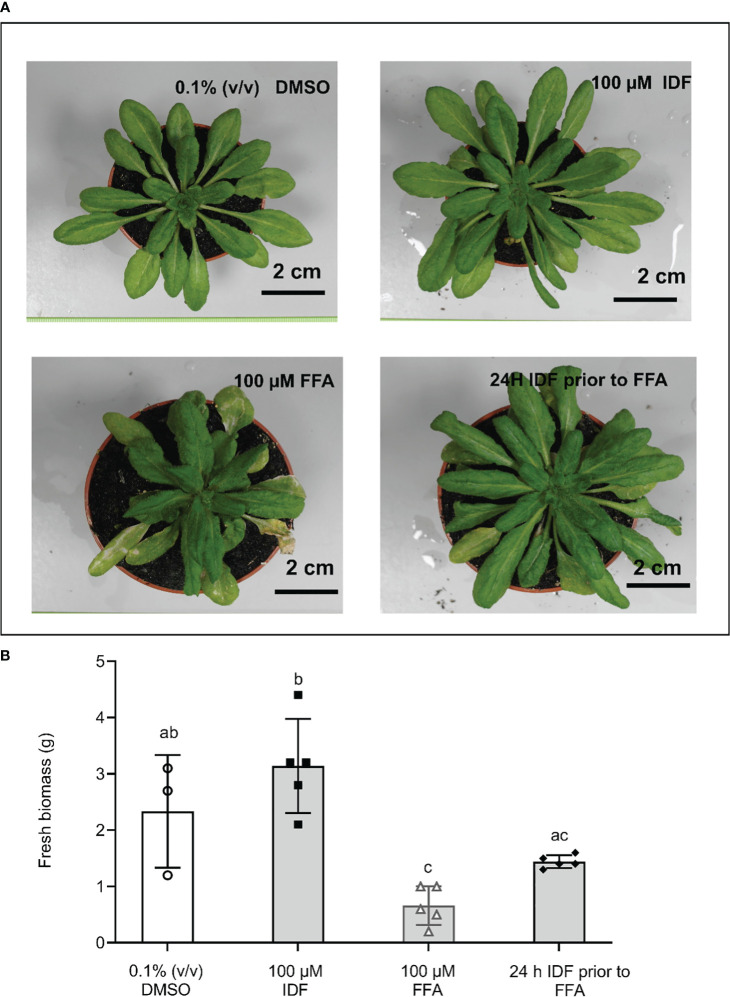
The pre-treatment of isoxadifen had a moderated protective effect against flufenacet toxicity in *Arabidopsis*. 3-week-old *Arabidopsis thaliana* ecotype Columbia-0 (Col-0) were treated with either 0.1% (v/v) DMSO, 100 µM IDF, 100 µM FFA, or pre-treated with 100 µM IDF for 24 h before the application of 100 µM FFA. **(A)** The photographs of whole rosettes were taken 14d after treatment. **(B)** The fresh biomass (FM) of individual rosettes from each treatment were determined after photographs were taken. Each bar represents an average FM of three to five biological replicates (mean ± SD, n =3-5) for each treatment. The symbols in each bar represent FM of individual rosettes. FM was compared using one-way ANOVA followed by turkey’s Posthoc test. Different letters represent significant differences among treatments (p ≤ 0.05).

FFA is commercially used without safener. While the GSTs that potentially detoxify FFA in monocot species have recently been identified (Ducker et al., 2019a; Ducker et al., 2019b; Ducker et al., 2020), the GSTs that detoxify FFA in *Arabidopsis* are currently unknown. To determine if the induction of *AtGSTUs* in the roots resulted in functional safening, we examined the toxicity of flufenacet in *Arabidopsis* plants treated with and without IDF. In addition, we examined the toxicity of *S*-MOC in *Arabidopsis* as a comparison. After 14 days of treatment, we observed an increased fresh biomass (FM) in IDF-treated plants compared to those in the control treatment (**Figures 5A, B**). While the FM of plants treated with FFA was significantly reduced compared to those in control group (**Figure 5B**, one-way ANOVA, p = 0.03), *S-*MOC treatment had no significant negative impact on biomass (**Supplementary Figure 8B**, one-way ANOVA, p = 1.00). Although IDF pre-treatment gave no significant protection to plants exposed to *S*-MOC (**Supplementary Figure 8B**, one-way ANOVA, p = 0.66), the safener pre-treatment suppressed the inhibition in rosette growth caused by FFA (**Figure 5B**, one-way ANOVA, p = 0.03). These results therefore confirmed that the enhancement of FFA detoxification in Arabidopsis roots correlated to a protective safeneing effect which was dependent upon the IDF being applied 24 h prior to FFA treatment.

### Selective activity of recombinant *At*GSTUs toward flufenacet

Taken together, the safener-inducible expression of the *AtGSTUs*, the enhanced metabolism of FFA by *S-*glutathionylation and the safening effects observed at the phenotypic level indicated that these are the promising candidate enzymes for safener-induced FFA detoxification in *Arabidopsis*. To examine enzyme activity toward FFA, the coding sequence (CDS) of each gene was used to express the respective recombinant proteins in *E. coli*. The glutathione conjugating activity toward the model substrate 1-chloro-2,4-dinitrobenzene (CDNB) and the herbicides FFA and *S*-MOC of each recombinant protein were then determined. In each case the enzymes were first assayed as crude lysates, with the protein extract from uninduced *E. coli* (-IPTG) used as a control (**Table 1**). All three *At*GSTUs (+IPTG) conjugated GSH to CDNB, with *At*GSTU24 at the highest (90 nkat mg ^-1^total lysate protein and *At*GSTU7 at the lowest rate (4 nkat mg ^-1^ total lysate protein). *At*GSTU19 lysates showed the highest rate of GSH conjugation toward *S*-MOC (~3 nkat mg ^-1^ total lysate protein) with *At*GSTU7 (~1.6 nkat mg^-1^ total lysate protein) showing significantly lower activity toward this herbicide. In contrast, lysates containing *At*GSTU7 showed a significantly high activity toward FFA (259 nkat mg ^-1^ total lysate protein) compared with *At*GSTU19 (2 nkat mg ^-1^ total lysate protein), or *At*GSTU24 (11 nkat mg ^-1^ total lysate protein). Based on the use of crude lysates direct comparison of the specific activities of the respective enzymes was not possible. However western blotting of the lysates with anti-GST-serum (**Supplementary Figure 9**), confirmed that all enzymes were expressed at broadly similar levels, and as such, the order of magnitude greater activity of *At*GSTU7 toward FFA provided the first indication of a key role for this enzyme in FFA detoxification in *Arabidopsis* root tissues.

**Table 1:** The specific activity of recombinant *At*GSTU7, *At*GSTU19 and *At*GSTU24 toward the model xenobiotic 1-chloro-2,4-dintrobenzene (CDNB) or herbicides flufenacet (FFA) and *S*-metolachlor (*S*-MOC).

Recombinant protein	Activity toward CDNB (nkat mg^-1^ purified protein)		Activity toward FFA (nkat mg^-1^ purified protein)		Activity toward *S*-MOC (nkat mg^-1^ purified protein)	
	-IPTG	+IPTG	-IPTG	+IPTG	-IPTG	+IPTG
***At*GSTU7**	0	5.27 ± 0.83**^a^**	2.89 ± 0.99	259.93 ± 18.45	1.14 ± 0.22	1.62 ± 0.48
***At*GSTU19**	0	30.74 ± 7.96^b^	0.78 ± 0.06	2.20 ± 0.40	0.69 ± 0.12	2.84 ± 0.24
***At*GSTU24**	13.44 ± 1.53	91.29 ± 5.88^c^	2.37 ± 0.09	10.63 ± 2.25	2.00 ± 0.99	2.90 ± 0.09

Crude lysates from *E. coli* expressing each of the GSTUs were assayed for product formation and activities reported as activity (nkat) per mg of total protein. The lysates from uninduced protein expression culture (-IPTG) were used as control. The data represent the mean of three independent replicates (mean ± SD, n = 3).

To further explore the selective activity toward FFA, the recombinant enzyme *At*GSTU7, along with *At*GSTU19, the enzyme most active toward the herbicide *S*-MOC, were affinity purified using their Strep-tags from the respective crude protein lysates (**Supplementary Figure S9**). The purified recombinant enzymes were subjected to kinetic analysis. The K_m_ values for each enzyme toward FFA were almost identical, suggesting the affinity for enzymes toward the herbicides were very similar. In contrast, the V_max_ for *At*GSTU7 was seven-fold higher and the turnover number for *At*GSTU7 (K_cat_) was approximately 9-times greater than those of *At*GSTU19 (**Table 2**). These results further confirmed the potential importance of *At*GSTU7 in FFA detoxification in *Arabidopsis* with its selective activity being due to enhanced turnover, as opposed to altered affinity for the herbicide.

**Table 2:** The kinetic analysis of purified recombinant *At*GSTU7 and *At*GSTU19 proteins toward herbicide flufenacet (FFA).

Enzyme	V_max_ (µmol min^-1^)	K_m_ (µmol)	K_cat_ (s^-1^)
***At*GSTU7**	0.14	109.57	231.64
***At*GSTU19**	0.02	95.08	25.12

The analysis of Michaelis constant to determine the affinity of enzyme to the substrate (K_m_), substrate turnover (K_cat_) and the maximum velocity of the catalytic reaction (V_max_) for AtGSTU7 and AtGSTU19 recombinant protein were analyzed. The value represented an average of three indepedent replicates (n = 3).

### The mutation of *At*GSTU7 significantly reduced flufenacet metabolism in *Arabidopsis* root tissues

To further confirm the role of *At*GSTU7 in FFA metabolism in *Arabidopsis*, the T-DNA insertion mutant of *AtGSTU7* (*gstu7*) as described in Ugalde et al. (2021) was used. FFA metabolism studies and transcript expression analyses were then performed with *gstu7* root tissues in comparison with those derived from wild-type (WT, ecotype Columbia-0, SALK_086642) *Arabidopsis*. For consistency, the *gstu7* mutant line was generated from the same WT background (Alonso et al., 2003). For both experiments, mutant and WT root tissues were treated with FFA alone as well as with IDF 24h prior to herbicide application.

**Figure 6:**
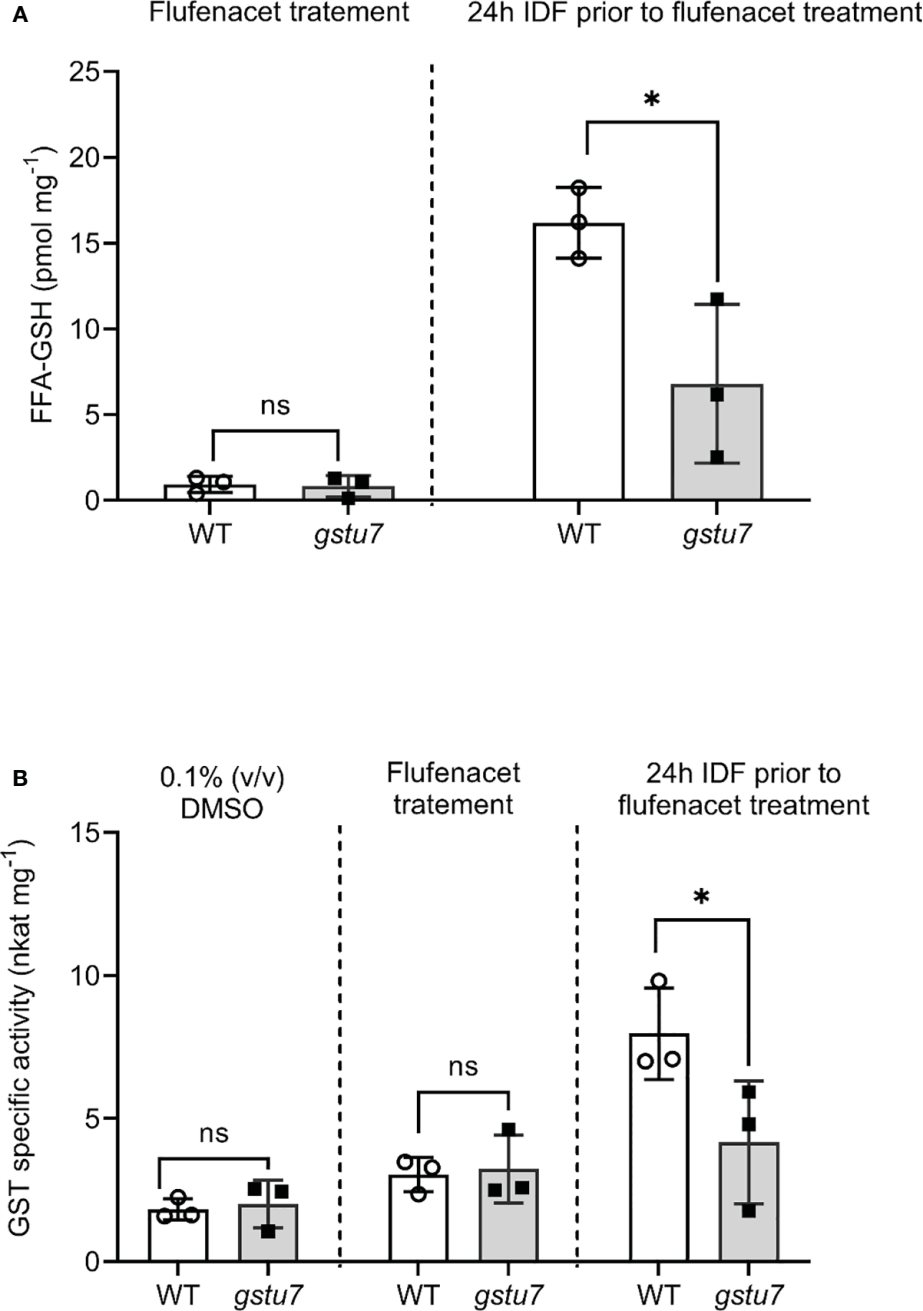
The reduction of flufenacet metabolisms in the *AtGSTU7* T-DNA insertion mutant (*gstu7*). Root tissues of wild type (WT) and gstu7 were treated with either flufenacet (FFA) alone, or FFA following a 24 h pre-treatment with isoxadifen (IDF). **(A)** The formation of the FFA glutathione conjugate and **(B)** the extractable GST activity toward FFA in WT and gstu7 root tissues were determined. Each bar represents an average concentration of FFA-GSH conjugate or GST activity of three biological replicates (means ± SD, n= 3) in WT or gtsu7 roots. The symbols in each bar represent the concentration of individual samples. The concentrations or the GST activity were compared between WT and gstu7 using Student’s t-test; asterisks indicate significant differences; * p ≤ 0.05.

Treatment with FFA alone did not lead to significant differences in the levels of FFA-GSH between the *gstu7* and WT root cultures (**Figure 6A**, Student’s *t*-test, p = 0.83). In contrast, in the IDF pre-treated plants, the amount of FFA-GSH formed in the WT root tissues was significantly higher (3 times) than in the *gstu7* root tissues (**Figure 6A**, Student’s *t*-test, p = 0.03). A similar pattern of elicitation was observed for extractable GST activities toward FFA from the crude extracts of WT vs. *gstu7* root tissues, with comparable activity levels were determined for WT and *gstu7* following exposure to FFA alone (**Figure 6B**, Student’s *t*-test, p = 0.82), while the 24 h pre-treatment of IDF resulted in a significant increase of GST activity toward FFA in WT vs. *gstu7* root tissues (**Figure 6B**, Student’s *t*-test, p = 0.04). These results confirm that *At*GSTU7 plays a major role in the IDF-induced enhanced glutathionylation of FFA.

### *In silico* modeling of *At*GSTU7

**Figure 7:**
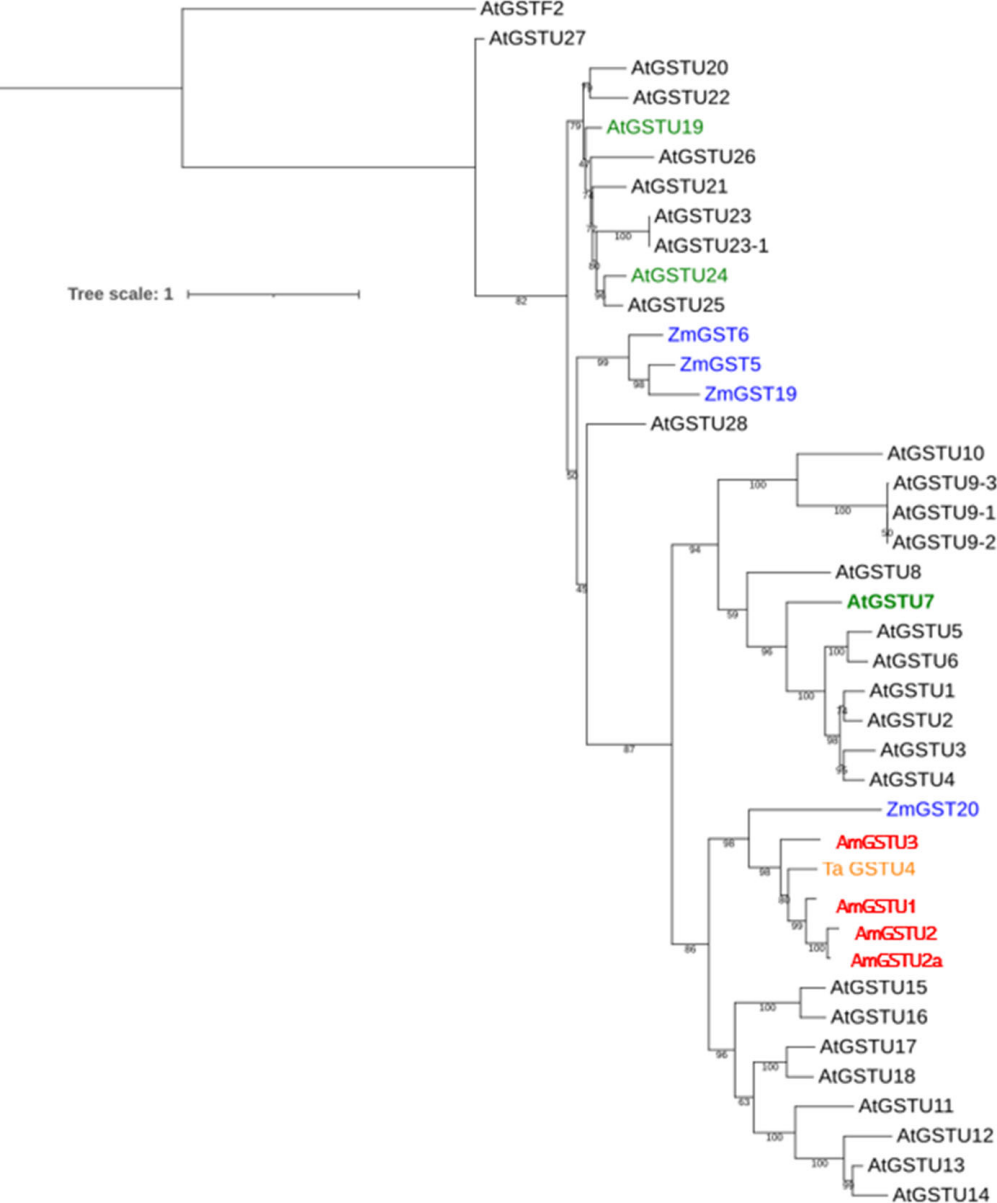
The phylogenetic analysis of *At*GSTU7 aminoacid sequences. The phylogenetic analysis of tau (U) GST family (GSTUs) from Arabidopsis (black), maize (blue), wheat (orange) and blackgrass (red). *Arabidopsis* GSTUs induced by IDF (*At*GSTU7, *At*GSTU19 and *At*GSTU24) are shown in green. The sequences of *Zm*GST5, *Zm*GST6, *Zm*GST19 and *Zm*GST20 (Irzyk and Fuerst, 1993; Dixon et al., 1998; McGonigle et al., 2000) wheat *Ta*GSTU4 (Thom et al., 2002) and blackgrass (*Alopecurus myosuroides*) *Am*GSTU1, *Am*GSTU2, *Am*GSTU2a and *Am*GSTU3 (Ducker et al., 2020; Franco-Ortega et al., 2021) were obtained from NCBI website (https://www.ncbi.nlm.nih.gov/).

The above findings as well as the differential activities of *At*GSTU7 vs. *At*GSTU19 and *At*GSTU24 on CDNB and FFA prompted us to investigate differences in the structure/activity characterisitics of the respective proteins. As a first step, we created a phylogenetic tree of *Arabidopsis* GSTUs based on publicly available sequence data (www.arabidopsis.org). As expected, *At*GSTU7 clustered separately from the monophyletic group containing *At*GSTU19 and *At*GSTU24 (**Figure 7**). While little is known about the family of GSTs that have activity toward FFA, a recent study in *Alopecurus myosuroides* (blackgrass) suggested six GST enzymes, notably (*Am*GST1 (tau), *Am*GST2 (tau), *Am*GST3 (tau), *Am*GST4 (phi), *Am*GST5 (phi) and *Am*GST6 (theta) as candidates for FFA detoxification based on their association with metabolism-linked resistance to the herbicide (Ducker et al., 2020). However, neither these blackgrass GSTs, or those from cereals linked to herbicide metabolism appeared related to the three *At*GSTUs (**Figure 7**).

**Figure 8:**
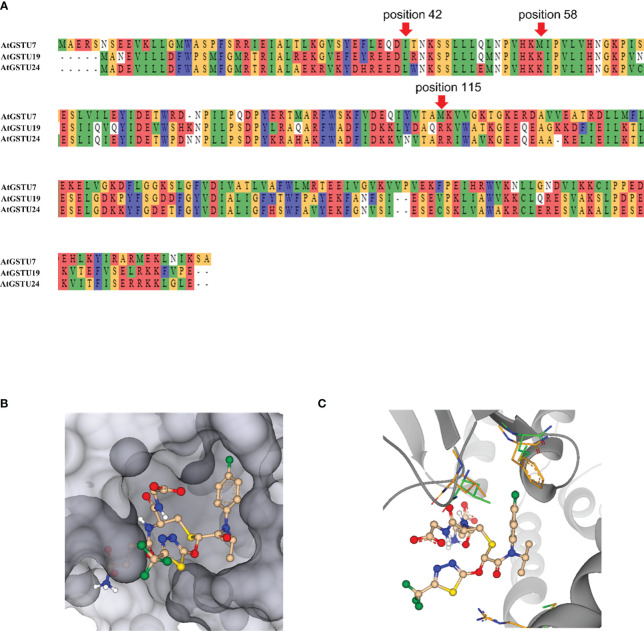
3D modeling of *At*GSTU7. **(A)** The amino acid sequence alignment of *At*GSTU7, *At*GSTU19 and *At*GSTU24. **(B)** A molecule mimicking the adduct of flufenacet fitted into the binding pocket of the *At*GST. While the thiadiazol points to the outside, the chlorphenylamide moiety binds into a protein pocket. **(C)** A molecule mimicking the adduct of GST flufenacet fitted into the binding pocket of the *At*GST. The amino acids at position 15, 42, and 115 which may make the main difference for flufenacet binding are shown in green for *At*GSTU7 and in orange for *At*GSTU24. *At*GSTU7 is visualized as dark-grey ribbon.

Multiple alignments showed that *At*GSTU19 and *At*GSTU24 amino acid sequences were 55% and 42% identical to those of *At*GSTU7 (**Figure 8A**). To probe whether this difference at amino acid sequence could contribute to the different functions, 3D protein structure models for the *At*GSTUs were created. The adduct of flufenacet bound to GST was modeled into a closely related 3D protein structure, *Ta*GSTU4-4 from wheat (Thom et al., 2002) and compared with structures predicted by Alphafold2 (AF2) for *At*GSTU7 (AF-Q9ZW24-F1-model), *At*GSTU19 (AF-Q9ZRW8-F1-model) and *At*GSTU24 (AF-Q9SHH6-F1-model) (Jumper et al., 2021; Varadi et al., 2022). The adduct was built assuming that the thiol moiety of the glutathione co-substrate attacks the FFA as the nucleophile, with the thiadiazol leaving group being readily released into the solvent (**Figure 8C**). Based on this orientation, the chlorphenylamide moiety of the herbicide would then point into a pocket formed by the amino acids lining the active site defined by amino acid positions namely M15, A17, S18, P19, P20, I42, M115, W167, Y214, R218 (all numberings according to the positions in the *At*GSTU7 protein).In contrast to the glutathione binding pocket, the amino acids contributing to substrate binding were more conserved. It is interesting that the sequence identities of the binding pocket ranged between 10% - 50% for individual pairings of enzymes, suggesting that even within a related phylogenetic clade, each *At*GST may have very different substrate specificities.

Wtihin the active sites of GSTs most amino acids stabilize GSH binding by either forming H-bonds, such as R23, K45, E71 and S72, or by providing hydrophobic pockets such as F20, I59 and P60. Generally, most *At*GSTs have a lysine (K) at position 58, or an amino acid capable of forming a H-bond to the amide carbonyl of glutathione, thereby stabilizing it in its bound conformation. The same carbonyl can be additionally stabilized by H-bonding to the amino acid at position 115, which in most *At*GSTs is an arginine (R). While *At*GSTU19 and *At*GSTU24 had lysine and arginine at position 58 and 115, respectively, *At*GSTU7 has methionine (M) in both positions (**Figure 8B**) which suggest that *At*GSTU7 cannot stabilize the carbonyl group of glutathione by H-bonding. This lack of H-bond stabilization would allow more conformational flexibility of the glutathione molecule within the active site, thereby potentially significantly broadening its substrate specificity toward different electrophilic co-substrates.

In the absence of crystal data for the bound complex, we are unable to pinpoint which amino acids define the binding of FFA. However, it is reasonable to propose that the difference at position 15 between the rotationally restricted and bulky phenylalanine (F) of *At*GSTU19 and *At*GSTU24, as compared with the much more flexible methionine in *At*GSTU7 would facilitate FFA binding based on the models. Similarly, the difference between the smaller isoleucine (I) at position 42 of *At*GSTU7, as compared to the leucine (L) in *At*GSTU19 and *At*GSTU724 would also widen the herbicide binding pocket to assist docking with FFA (**Figures 8B, C**). Collectively, we propose that the more accessible herbicide binding site in combination with a less conformationally restricted GSH co-substrate could account for the greater activity of *At*GSTU7 toward flufenacet, as compared with *At*GSTU19 and *At*GSTU24.

## Discussion

Despite their remarkable activity in monocot crops, it remains elusive why safeners are unable to protect dicot crops from herbicide damage. In this study, we shed new light on the safener response of *Arabidopsis*, a model dicotyledonous species. The current paradigm suggests that the ability of safeners to protect plants from herbicide injury is restricted to cereals and does not extend to broad leaf dicotyledonous plants (Kraehmer et al., 2014). Here, we report that a restricted protective response to the herbicide flufenacet can be invoked by the safener isoxadifen in *Arabidopsis*. Several studies have previously demonstrated that GSTs can be induced in *Arabidopsis* by a range of safener chemistries, including benoxacor, isoxadifen, mefenpyr, and fenclorim (DeRidder et al., 2002; Brazier-Hicks et al., 2008; Behringer et al., 2011). Our study demonstrates that IDF triggers the localized transient induction of several GSTs transcripts in root tissues. As such, at the level of transcriptional regulation, the safener responsiveness of *Arabidopsis* is remarkably similar to that determined in cereals such as maize or rice (Brazier-Hicks et al., 2020; Brazier-Hicks et al., 2022). This confirms that the ability to recognize safeners and respond to them through rapid gene activation of protective detoxification genes is conserved between cereals and dicotyledonous species. Furthermore, our studies confirm that in the case of GST-mediated detoxification, the induction of the respective genes is also associated with the enhanced expression of functional detoxifying enzymes. However, the results of our study highlight several factors that contributing to a lack of effective safening of dicot plants as encountered in agricultural practise:

Firstly, the safener response in *Arabidopsis* observed in our experimental system was predominantly restricted to the root tissues. The magnitude of the enhancement of *AtGSTU7*, *AtGSTU19*, and *AtGSTU24* was much greater in roots than in rosettes, indicating a more restrained localized induction of genes in *Arabidopsis* than in rice or maize (DeRidder and Goldsbrough, 2006). Secondly, the different GST spectrum in *Arabidopsis* has a significant influence on the outcome on the protective effects of safeners. We demonstrated that FFA-detoxification was primarily associated with a single enzyme *At*GSTU7. This is in contrast to the case in cereals where multiple GSTs can often detoxify a single herbicide (Dixon et al., 1998). In common with the herbicide detoxifying GSTs in cereals, *At*GSTU7 is known to have a cytosolic localization in *Arabidopsis* (Dixon et al., 2009), where it is well placed to utilize cellular glutathione in the rapid conjugation of absorbed toxic xenobiotics.

Furthermore, in contrast to monocot crops, significant safening effects in *Arabidopsis* were only observed after a 24 h pre-exposure to IDF. This is different to cereals, where the protective response can be achieved by co-application of safener and herbicide (Riechers et al., 2010). Interestingly, the transcript induction studies showed that the greatest enhancement in *At*GSTU expression was seen when IDF was pre-treated for 24 h followed by a 1 h exposure to FFA. Overall, a pre-exposure to IDF would appear to be required to achieve sufficient levels of transcript induction, enzyme activity and enhancement of FFA metabolism, eventually leading to the observed protective or ‘safening’ effects against FFA in *Arabidopsis*.

The safener-induced detoxification of FFA in *Arabidopsis* was strongly linked to the upregulation and activity of *At*GSTU7, as confirmed using the respective T-DNA insertion mutant line. Thus, the studies with the *gstu7* knockout showed that the IDF-induced GST activity toward FFA was much reduced as compared with wild type plants, with the residual conjugation being presumably due to the presence of the other less active conjugating enzymes such as *At*GSTU19 and *At*GSTU24. *At*GSTU7 transcript expression was strongly induced (> 300-fold) early (1 h) after FFA treatment in root tissues pre-treated with IDF for 24 h prior to FFA treatment. This inducibility was then matched by an unusual enzyme specificity toward FFA, with *At*GSTU7 having a 10-fold higher catalytic capacity toward the herbicide as compared to *At*GSTU19 (**Table 2**). In this particular scenario, the strong induction of *AtGSTU7*, matched by its ability to detoxify a specific herbicide then provided a classic safener response. FFA is known to be detoxified by multiple GSTUs in wild grasses and cereals (Ducker et al., 2019a; Ducker et al., 2019b; Ducker et al., 2020), though the respective GSTUs responsible are not closely related to *At*GSTU7 (**Figure 8A**). Structural modeling studies of *At*GSTU7 suggested that the high activity of the enzyme toward this herbicide could be explained by the less constrained binding of the thiol donor glutathione, coupled to a more accessible hydrophobic binding site. While this can only be confirmed by future co-crystallisation studies, this explanation is in line with the observed kinetic characteristics of *At*GSTU7 toward FFA (**Table 2**).

## Conclusion

In our study, we provide the first evidence of effective safener-induced herbicide detoxification in dicots, as demonstrated for the model dicot *Arabidopsis thaliana*. Through our analysis, we could demonstrate the novel activity of safener-inducible *At*GSTU7, playing a central role in FFA detoxification. However, this could only be observed after pre-treatment with the safener IDF, indicating a major difference between safening in dicots and monocot crops. In addition, IDF-induced detoxification of the herbicide FFA could only be observed in root tissue of *Arabidopsis*, suggesting that tissue-specific restriction of safener activity may be another factor contributing to differences in safener activity between dicots and monocots.

This study highlights the potential to partner safener-inducible enzymes with herbicides that can be readily detoxified by them as a route to new safener applications in dicotyledonous species. The better understanding of the differences between monocots and dicots with respect to safener activity may provide the basis for further research into developing effective herbicide-safener combinations for dicot crops.

## Data availability statement

The original contributions presented in the study are included in the article/**Supplementary Material**. Further inquiries can be directed to the corresponding authors.

## Author contributions

GP-C: Data curation, Formal analysis, Investigation, Methodology, Writing – original draft. NO: Data curation, Formal analysis, Project administration, Supervision, Validation, Writing – original draft, Writing – review & editing. AG-C: Formal analysis, Investigation, Software, Writing – original draft. GL: Formal analysis, Investigation, Methodology, Software, Visualization, Writing – original draft. JD: Conceptualization, Funding acquisition, Investigation, Project administration, Resources, Supervision, Validation, Writing – review & editing. RE: Conceptualization, Funding acquisition, Project administration, Resources, Supervision, Validation, Writing – review & editing.
