# Supplementary material for: Selective herbicide safening in dicot plants: a case study in Arabidopsis
Source: Front Plant Sci. 2024 Jan 15;14:1335764. doi: 10.3389/fpls.2023.1335764 (PMC10822893; doi:10.3389/fpls.2023.1335764)
Supplement: Supplementary file 1 [file Presentation_1.pdf]

frontiers

Supplementary Material

Selective herbicide safening in dicot plants: a case study in *Arabidopsis*

Gabriela Pingarron-Cardenas[1#], Nawaporn Onkokesung[1], Alina Goldberg-Cavalleri[1], Gudrun Lange[2], Jan Dittgen[3]†[*], Robert Edwards[1]†[*]

†These authors contributed equally to this work and share last authorships

[1]Agriculture, School of Natural and Environmental Sciences, Newcastle University, Newcastle Upon Tyne, UK

[2] Bayer AG, Crop Science Division, Computational Life Sciences, Frankfurt, Germany

[3] Bayer AG, Crop Science Division, Weed Control Research, Frankfurt, Germany

[#] Present Address: Department of Plant Sciences, University of Cambridge, Cambridge, UK.

*** Corresponding authors:**

Robert Edwards:robert.edwards@newcastle.ac.uk, Jan Dittgen: jan.dittgen@bayer.com

Supplementary Tables

Supplementary Table S1 The calculated accurate mass of flufenacet and *S*-metolachlor in *Arabidopsis thaliana.* The parents and metabolites were extracted from root or rosette tissues of *Arabidopsis* at 24 h after treated with herbicide alone or herbicides and safener

**Compound**	**Calculated mass (*m/z*)**
**Flufenacet**	364.0743
**Flufenacet-glutathione conjugate (FFA-GSH)**	501.1819
**Flufenacet-glutamyl cysteine conjugate**	444.1605
**Flufenacet-cysteinyl glycine conjugate**	372.1393
**Flufenacet-cysteine conjugate**	315.1179
***S*-metolachlor**	284.1417
***S*-metolachlor-glutathione conjugate (*S*-MOC GSH)**	555.2489
***S*-metolachlor-cysteinyl glycine**	426.2063
***S*-metolachlor-glutamyl cysteine**	498.2274
***S*-metolachlor-cysteine**	369.1948

Supplementary Table S2 Primer sequences tested for transcript expression analysis in Arabidopsis root cultures and shoots. Genes shown in bold correspond to reference genes.

Gene ID	Accession Number	Sequence (5'- 3')
**SAND**	**AT2G28390**	**CAAGGCAGGAAATCACCAGG**
		**CCTCCAAGCAAGGGTGTCAT**
**GSTU7**	AT2G29420	ACTATGGCTCGATTCTGGTCT
		TCTCCTCCGTTCTCATCAGC
**GSTU19**	AT1G78380	GCTAGGTTCTGGGCTGATTTC
		AGCCAAAGTCATCGCCACTA
**GSTU24**	AT1G17170	GACTTGGCCCGACAATAACC
		AACTCCTTGGCTGCTTCTTG
**GSTU10**	AT1G74590	CGGTTCTGGGTCAGCTACAT
		ACACCGATTGCTTCACGATG
**GSTU11**	AT1G69930	CTGTGGCTCGATTTTGGGAC
		ACCCGATGTTTTCTCCTCCA
**GSTU26**	AT1G17190	GATGAGGTTTGGTCCGATGC
		TCACTGCTGCATGTTCTTCG
**GSTF2**	AT4G02520	GTGAGCACAAGAAGGAGCCT
		TGGCCATGATTGCGTACTGA
**GSTF8**	AT2G47730	CGGTCAAATTCCTGCTCTCG
		TGCCTTTAAAGACACGCTCG
**GSTL1**	AT5G02780	GATCAAGACTCTCCCCAGTGA
		AGCTACACTTGCCCATTTGC

Supplementary Figures

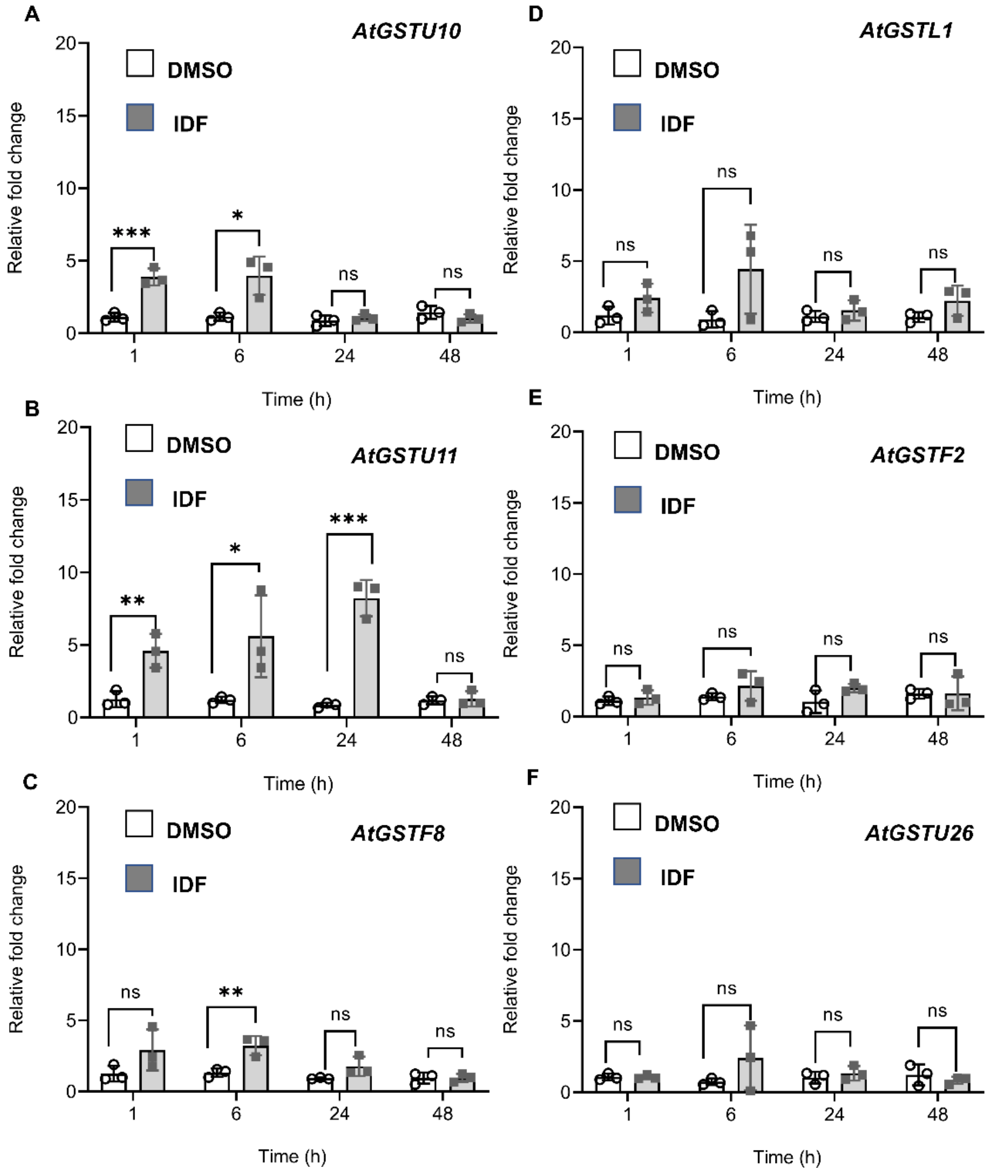

Supplementary Figure S1. The relative transcript expression of *Arabidopsis* glutathione-S-transferase Tau (U), Phi (F) and Lambda (L) families after isoxadifen safener treatment in root tissues**.** The relative transcript expression of (A) *AtGSTU10*, (B) *AtGSTU11*, (C) *AtGSTF8*, (D) *AtGSTL1*, (E) *AtGSTF2* and (F) *AtGSTU26*, at1 h, 6 h, 24 h and 48 h after isoxadifen (IDF) or solvent control (DMSO) treatment in root tissues of *Arabidopsis*. Each bar represented average of three biological replicates (mean ± SD, n =3) of relative fold change of each treatment at designated time point after treatment. The relative transcript expression (fold change) of each gene were compared between sample treated with solvent control (DMSO) and isoxadifen-ethyl using Student's *t*-test, asterisks indicate significant differences; * $p \leq 0.05$; ** $p \leq 0.01$; *** $p \leq 0.001$, ns = no statistic difference.

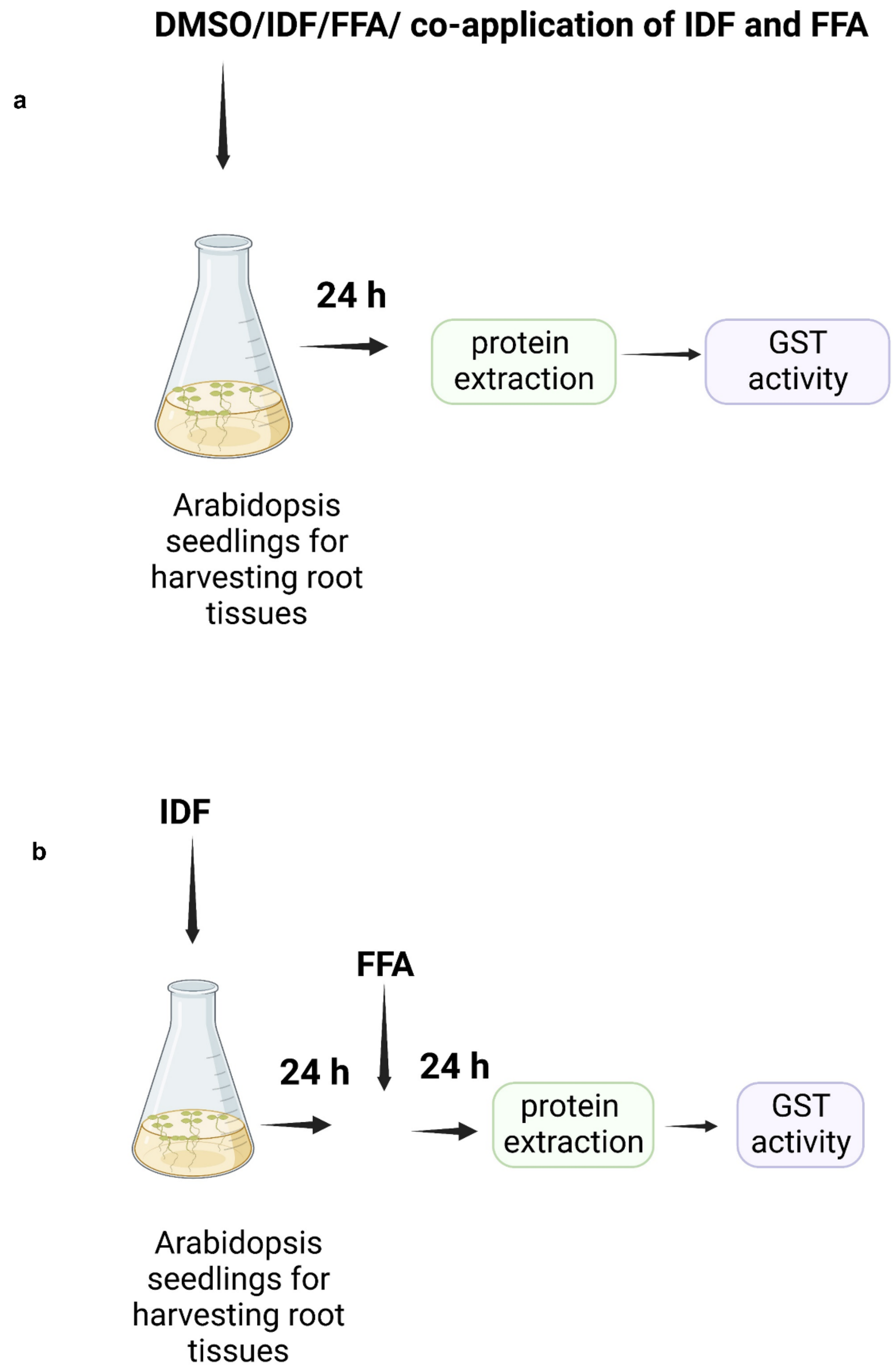

Supplementary Figure S2. Schematics of isoxadifen and herbicide treatments in root and rosette tissues of Arabidopsis from herbicide metabolites, transcript expression and GST activity toward herbicides.

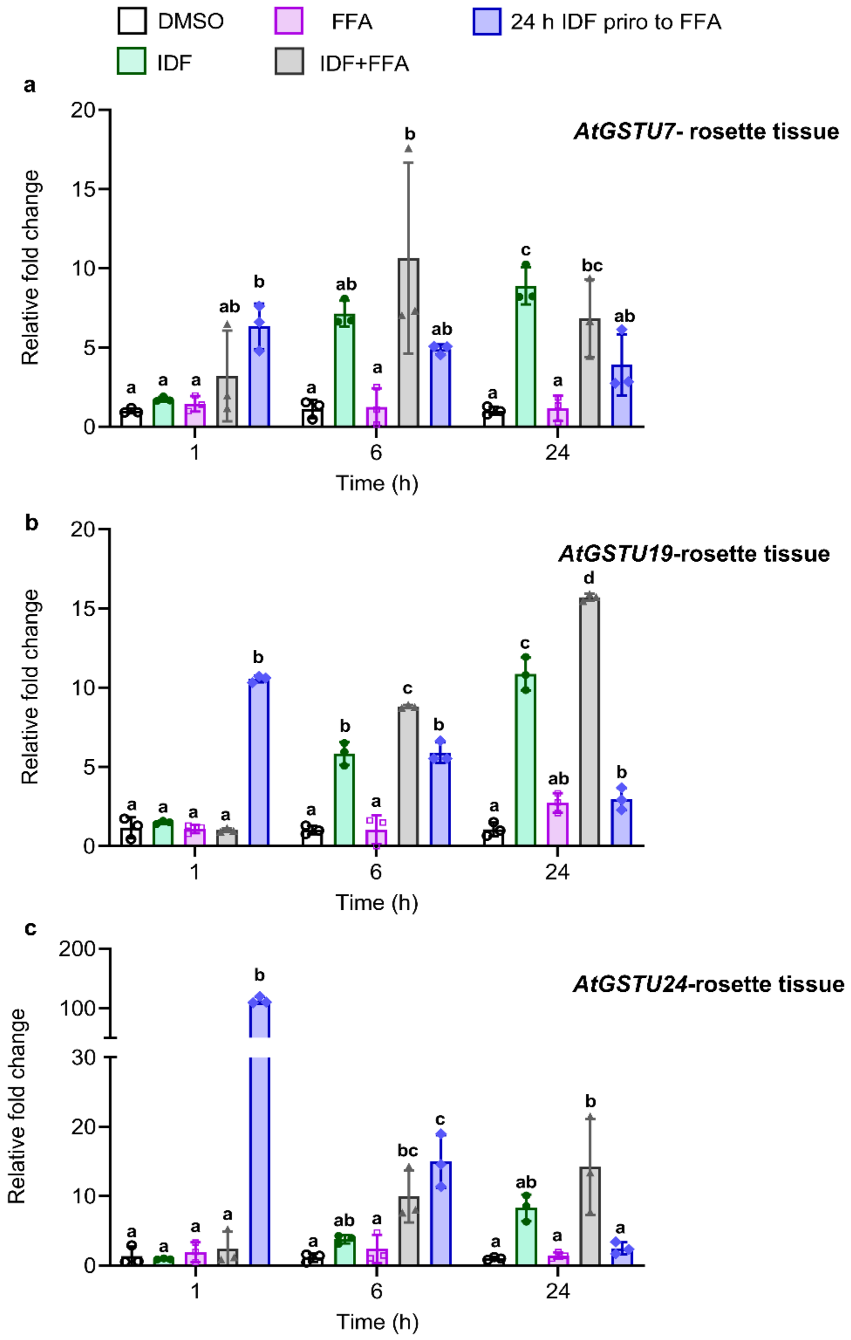

Supplementary Figure S3. The relative transcript expression of *AtGSTU7*, *AtGSTU19* and *AtGTSU24* in *Arabidopsis* rosette tissues. The excised rosette tissues from soil-grown *Arabidopsis* plants were treated with carrier solvent alone (DMSO), isoxadifen (IDF), flufenacet (FFA), co-application of IDF and FFA or pre-treatment of IDF for 24 h before an application of FFA. The relative fold change of (A) *AtGSTU7*, (B) *AtGSTU19* and (C) *AtGSTU24* were determined at designated time points. Each bar represents an average fold change of three biological replicates (means ± SD, n= 3) and the symbols in each bar represent fold change of individual sample. The relative fold changes were compared among the treatment using one-way ANOVA followed by turkey's *Posthoc* test, the different letter indicated significant differences; $p \leq 0.05$.

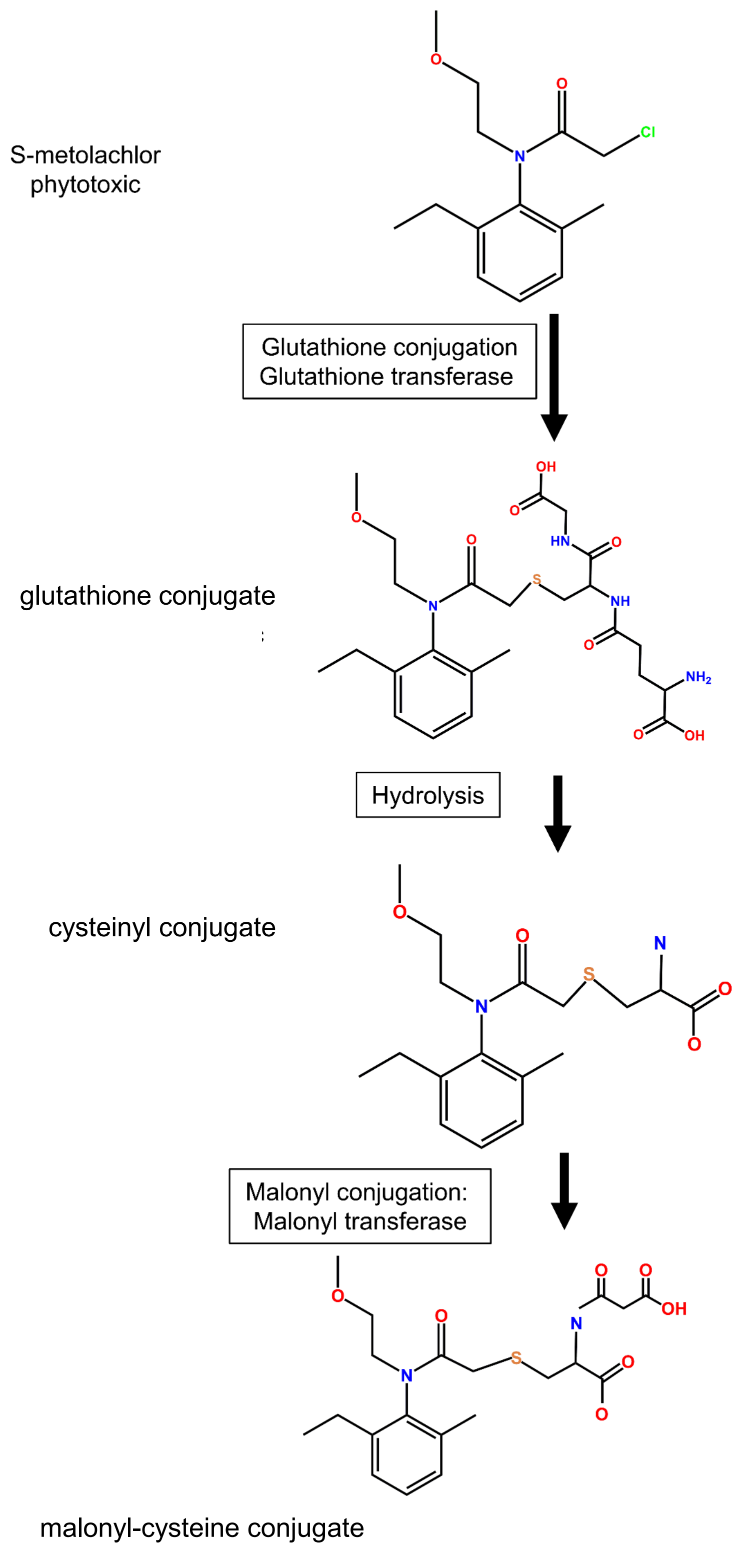

Supplementary Figure S4. *S*-metolachlor detoxification pathway in plants.

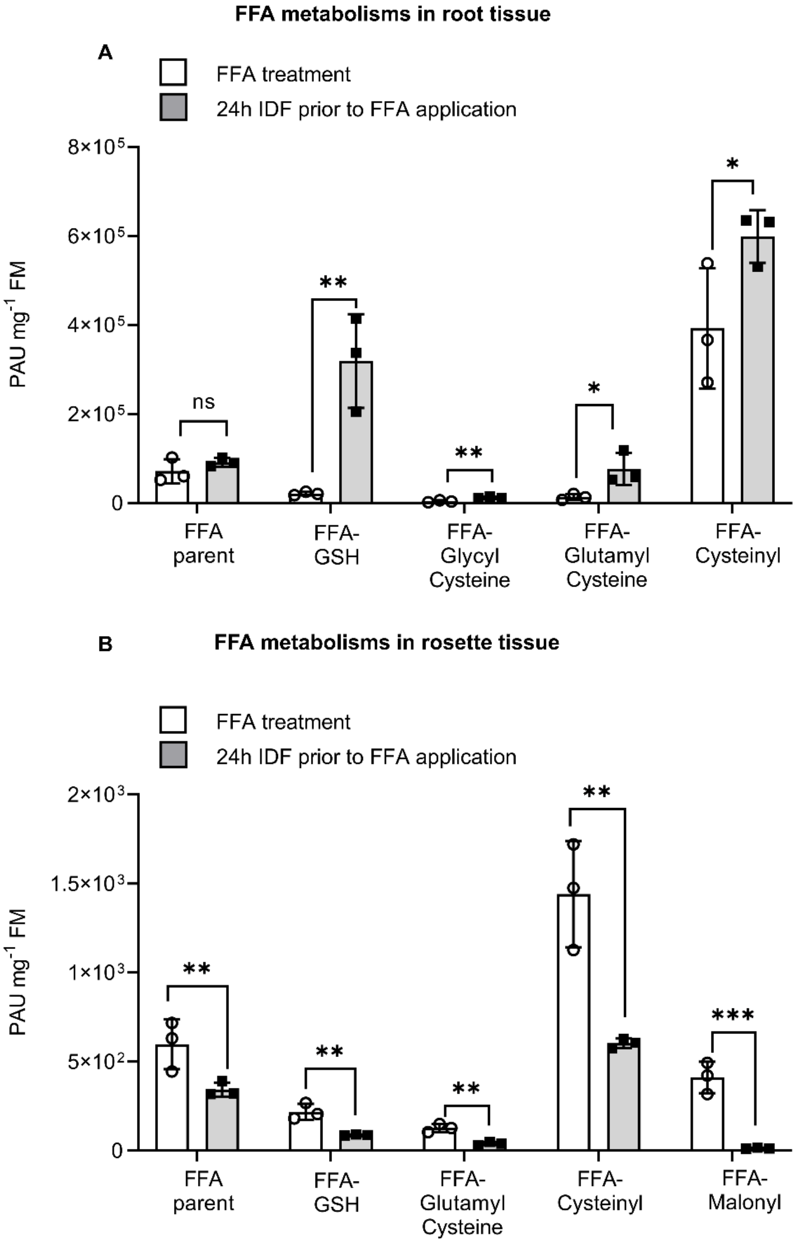

Supplementary Figure S5. The effect of isoxadifen treatment on flufenacet metabolisms in *Arabidopsis* root and rosette tissues. The formation of flufenacet (FFA) metabolites including glutathione (GSH)- , glycyl-cysteine-, glutamyl-cysteine-, malonyl- and cysteine-conjugates were quantified at 24 h after treatments of FFA alone or 24h pre-treatment of IDF before application of FFA in (A) root, or (B) rosettes tissues. The formation of metabolites was quantified based on peak area of each metabolite identified based on accurate mass. Each bar represented average of three biological replicates (mean ± SD, n =3) of FFA parents or FFA metabolites. The formation of FFA and its metabolites were compared between sample treated with FFA and 24 h pre-treatment with isoxadifen before application of FFA using Student's *t*-test, asterisks indicate significant differences; * $p \leq 0.05$; ** $p \leq 0.01$; *** $p \leq 0.001$, ns = no statistic difference.

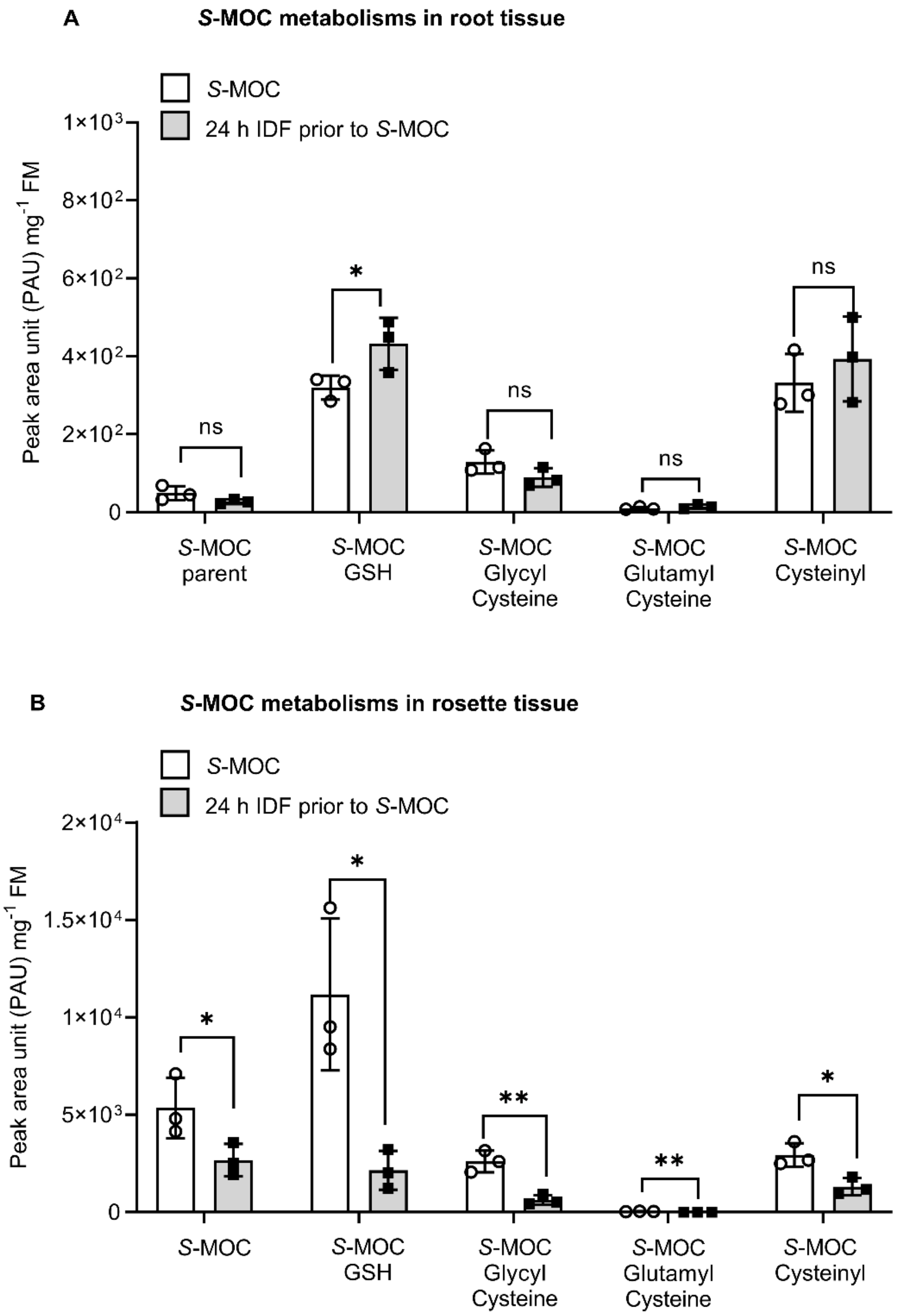

Supplementary Figure S6. The effect of isoxadifen treatment on *S*-metolachlor metabolisms in *Arabidopsis* root and rosette tissues. The formation of *S*-metolachlor (*S*-MOC) metabolites including glutathione (GSH)- , glycyl-cysteine-, glutamyl-cysteine-, and cysteine-conjugates were quantified at 24 h after treatments of *S*-MOC alone or 24h pre-treatment of IDF before application of *S*-MOC in (A) root, or (B) rosettes tissues. The formation of metabolites was quantified based on peak area of each metabolite identified based on accurate mass (Supplementary Table S1). Each bar represented average of three biological replicates (mean ± SD, n =3) of *S*-MOC parents or *S*-MOC metabolites. The formation of *S*-MOC and its metabolites were compared between sample treated with *S*-MOC and 24 h pre-treatment with isoxadifen before application of *S*-MOC using Student's *t*-test, asterisks indicate significant differences; * $p \leq 0.05$; ** $p \leq 0.01$; ns = no statistic difference.

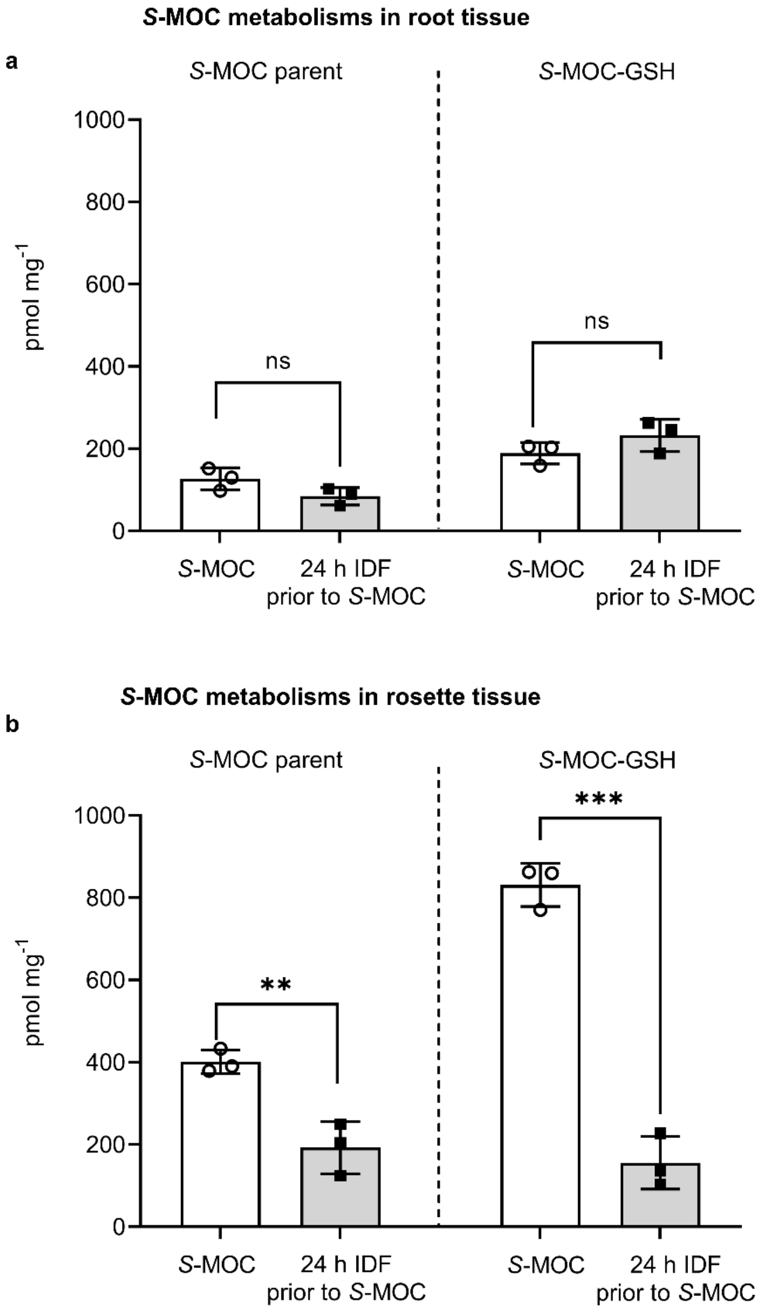

Supplementary Figure S7. The effects of isoxadifen treatment on *S*-metolachlor and glutathionylated *S*-MOC in Arabidopsis root and rosette tissues. The root cultures or rosettes of *Arabidopsis* were treated with safener: herbicide: S-metolachlor (*S*-MOC) or pre-treatment with IDF for 24 h followed by *S*-MOC. The levels of *S*-MOC (parent) and its glutathione-conjugated in (A) root culture and (B) excised rosettes were analysed after 24 h of treatments. Each bar represented average of three biological replicates (mean ± SD, n =3) of *S*-MOC parents or *S*-MOC metabolites. The formation of *S*-MOC and its metabolites were compared between sample treated with *S*-MOC and 24 h pre-treatment with isoxadifen before application of *S*-MOC using Student's *t*-test, asterisks indicate significant differences; * $p \leq 0.05$; ** $p \leq 0.01$; ns = no statistic difference.

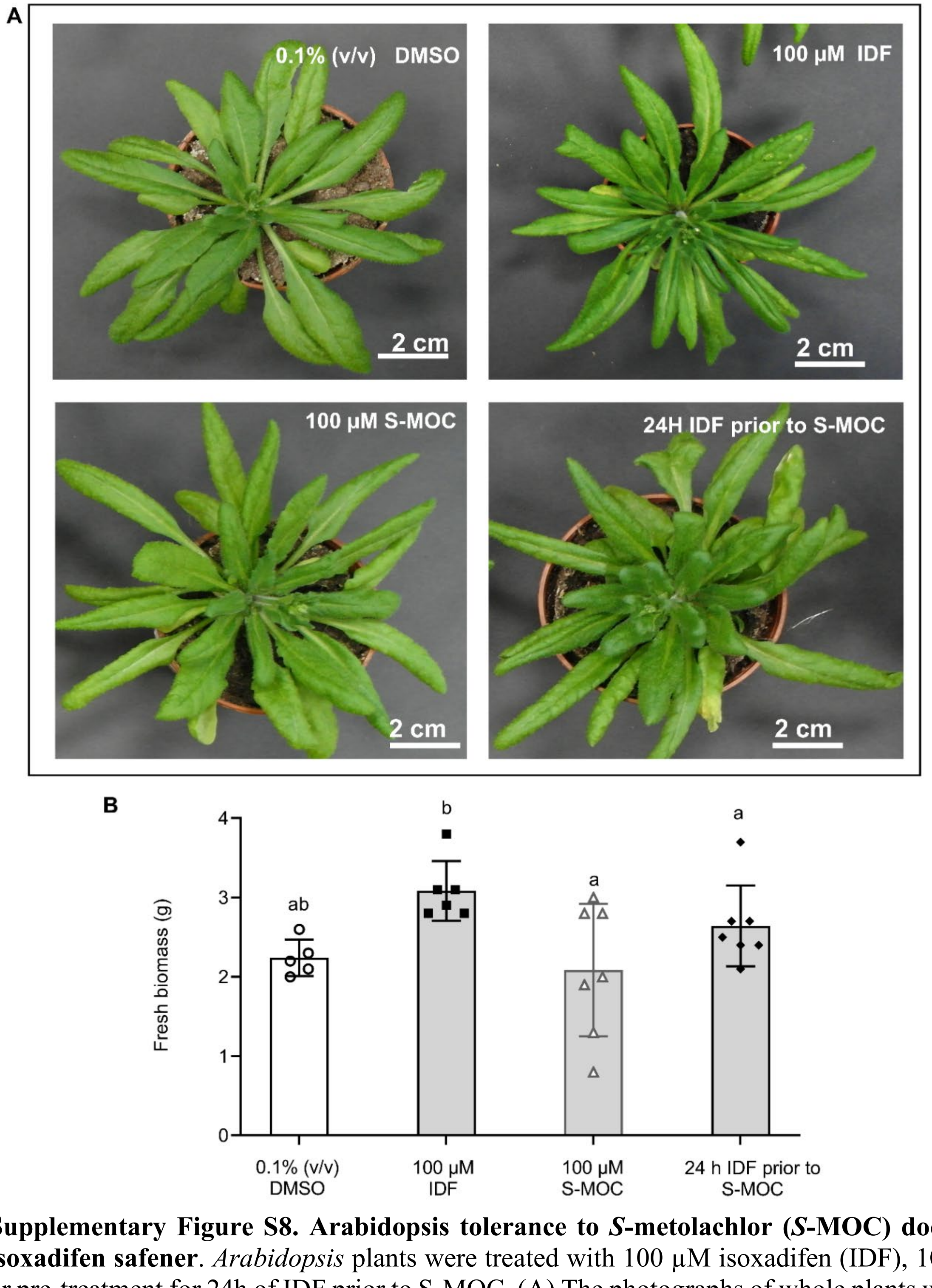

Supplementary Figure S8. Arabidopsis tolerance to *S*-metolachlor (*S*-MOC) does not require isoxadifen safener. *Arabidopsis* plants were treated with 100 µM isoxadifen (IDF), 100 µM *S*-MOC or pre-treatment for 24h of IDF prior to S-MOC. (A) The photographs of whole plants were taken after 14 d of treatment. (B) The fresh biomass (FM) of rosette tissues were determined at 14 d after treatment. Each bar represented average of three-five individual plants (mean ± SD, n =3-5) of FM at 14d after treatment. The average FMs were compared among treatment using one-way ANOVA followed by turkey's *Posthoc* test, the different letter indicated significant differences; $p \leq 0.05$.

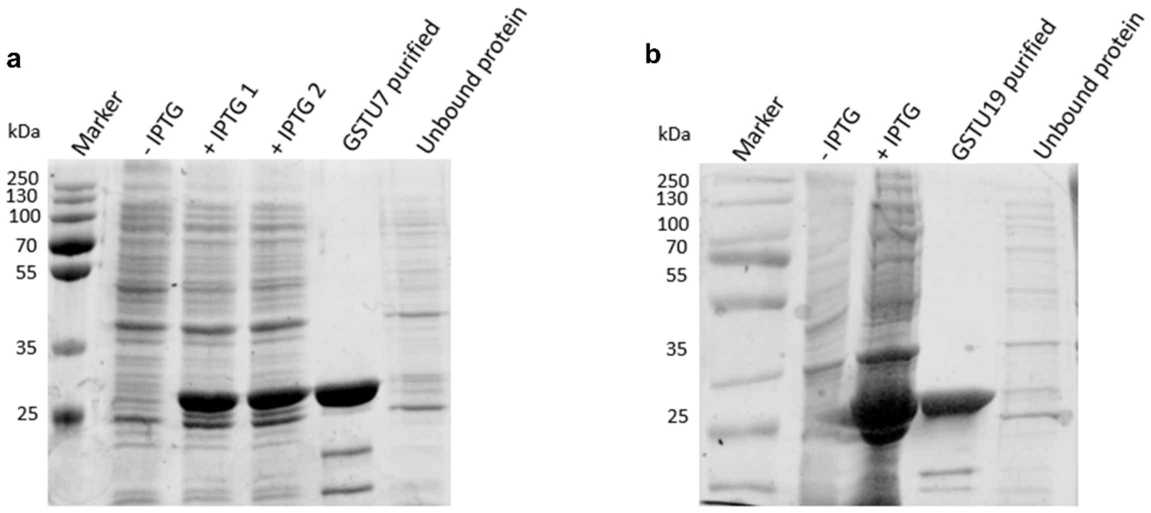

Supplementary Figure S9. Purified recombinant *At*GSTU7 and *At*GSTU19 proteins. The recombinant proteins were purified from *Escherichia coli* expressing Strep-tag fusion of (A) *At*GSTU7 or (B) *At*GSTU19.
